# How effective are organizational-level interventions in improving the psychosocial work environment, health, and retention of workers? A systematic overview of systematic reviews

**DOI:** 10.5271/sjweh.4097

**Published:** 2023-07-01

**Authors:** Birgit Aust, Jeppe Lykke Møller, Mads Nordentoft, Karen Bo Frydendall, Elizabeth Bengtsen, Andreas Brøgger Jensen, Anne Helene Garde, Michiel Kompier, Norbert Semmer, Reiner Rugulies, Sofie Østergaard Jaspers

**Affiliations:** 1 National Research Centre for the Working Environment, Copenhagen, Denmark.; 2 VIVE, The Danish Center for Social Science Research, Copenhagen, Denmark.; 3 Ascent Consulting Group, Copenhagen, Denmark.; 4 Department of Psychology, University of Copenhagen, Copenhagen, Denmark.; 5 Behavioural Science Institute, Radboud University, Nijmegen, The Netherlands.; 6 Department of Psychology, University of Bern, Bern, Switzerland.; 7 Biological Work and Health Psychology, University of Konstanz, Germany.; 8 Department of Public Health, University of Copenhagen, Copenhagen, Denmark.

**Keywords:** burnout, combined intervention, distal outcome, individual intervention, influence at work, proximal outcome, psychosocial work environment, working condition, working time intervention, workplace intervention

## Abstract

**Objective:**

This study aimed to systematically review the effectiveness of organizational-level interventions in improving the psychosocial work environment and workers’ health and retention.

**Methods:**

We conducted an overview of systematic reviews on organizational-level interventions published between 2000 and 2020. We systematically searched academic databases, screened reference lists, and contacted experts, yielding 27 736 records. Of the 76 eligible reviews, 24 of weak quality were excluded, yielding 52 reviews of moderate (N=32) or strong (N=20) quality, covering 957 primary studies. We assessed quality of evidence based on quality of review, consistency of results, and proportion of controlled studies.

**Results:**

Of the 52 reviews, 30 studied a specific intervention approach and 22 specific outcomes. Regarding intervention approaches, we found strong quality of evidence for interventions focusing on “changes in working time arrangements” and moderate quality of evidence for “influence on work tasks or work organization”, “health care approach changes”, and “improvements of the psychosocial work environment”. Regarding outcomes, we found strong quality of evidence for interventions about “burnout” and moderate quality evidence for “various health and wellbeing outcomes”. For all other types of interventions, quality of evidence was either low or inconclusive, including interventions on retention.

**Conclusions:**

This overview of reviews identified strong or moderate quality of evidence for the effectiveness of organizational-level interventions for four specific intervention approaches and two health outcomes. This suggests that the work environment and the health of employees can be improved by certain organizational-level interventions. We need more research, especially about implementation and context, to improve the evidence.

The psychosocial work environment is associated with a variety of health outcomes (1) and is considered as an arena in which interventions can reach a large number of employees through primary preventive activities on the organizational-level (2–4), ie, through changes in how work is organized and conducted.

When evaluating the effectiveness of organizational-level interventions, it is sensible to distinguish between effects on proximal and distal endpoints. Proximal endpoints relate to the effects of organizational-level interventions on conditions in the psychosocial work environment, eg, increasing workers’ influence or preventing exposure to violence at work. Distal endpoints concern the effects of organizational-level interventions on workers’ mental and physical health and retaining at work (see figure 1).

**Figure 1:**
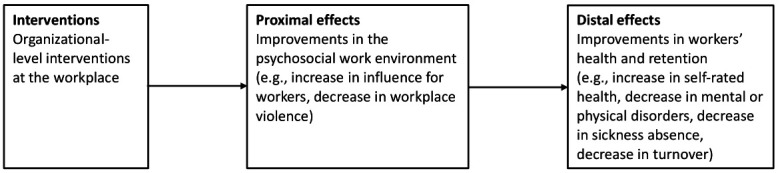
Proximal and distal effects of organizational-level interventions at the workplace.

Despite thousands of studies of interventions addressing the psychosocial work environment during the last decades, the effectiveness of these interventions is still unclear (5). Reviews have come to conflicting conclusions, ranging from suggesting no (6) or only limited effects (7), to concluding that organizational-level interventions are effective (8, 9).

Compared to reviews of primary studies, overviews of reviews, also termed “umbrella reviews” or “meta-reviews”, enable researchers examining the field from “higher up” and potentially identify patterns that can be difficult to detect in reviews of primary studies (10–12). To our knowledge, five overviews of reviews on organizational-level workplace interventions have been published in the last 15 years (13–17). However, none of them have included both proximal and distal effects of organizational-level interventions. Instead, these overviews of reviews focused on specific effects of the interventions on health (13), common mental disorders (17), stress (14), workplace absence (15), and workplace disability (16), respectively. Several of these reviews were also focused more on secondary and tertiary prevention than primary prevention.

Therefore, we set out to provide a comprehensive and updated overview of reviews, evaluating the effectiveness of organizational-level interventions on both proximal endpoints (changes in the psychosocial work environment) and distal endpoints (changes in workers’ health and wellbeing, and retention) with a focus on primary prevention.

## Methods

### Design and protocol

This is an overview of reviews on the effects of psychosocial work environment interventions. We structured the overview in accordance with the Preferred Reporting Items for Systematic Reviews and Meta-Analyses (PRISMA) statement (18). Although PRISMA was developed for reviews of primary studies rather than overview of reviews, most PRISMA items are applicable.

We conducted this overview of reviews in two stages. At stage one, we published in 2017 a Danish language report of the overview of reviews on the homepage of the Danish National Research Centre for the Working Environment (19). At stage two, we prepared the present manuscript for the international research community with an updated literature search until November 2020. (See the supplementary material www.sjweh.fi/article/4097, e-Appendix 1 for details.)

### Eligibility criteria

Organizational-level interventions were defined as interventions aimed at improving the psychosocial work environment through organizational-level changes, such as organizational policies, leadership style, or working conditions, or through improvement of competencies to handle work tasks. Workplace interventions that solely aimed at changing employees’ individual coping strategies (eg, teaching mindfulness techniques) were excluded.

The following inclusion criteria for eligibility of a review were applied: (i) systematic review (with or without meta-analyses); (ii) ≥1 study in the review was about a planned organizational-level intervention at the workplace with the aim of improving the psychosocial work environment and assessed either changes in the (a) psychosocial work environment, (b) workers’ health and wellbeing, or (c) retention. Reviews including both organizational-level and individual-based interventions had to provide distinct analyses or distinct conclusions for the organizational-level interventions; (iii) intervention effects were evaluated using quantitative methods. Reviews including also qualitative evaluations had to provide distinct analyses or conclusions for the quantitative effect evaluation; and (iv) reviews had to be in English and published in a peer-reviewed journal between January 2000 and 30 November 2020.

Reviews were excluded if the interventions were exclusively: (i) conducted outside the workplace; (ii) individual-based, ie, aimed to change behavior, thoughts, or feelings of employees without changes in the psychosocial work environment; (iii) directed towards employees with a defined disease or disorder (tertiary prevention); (iv) directed towards participants who were not employees (eg, students); or (v) concerned with the evaluation of economic effects or with changes in client or customer satisfaction.

### Search strategy

We conducted our search using three strategies: (i) systematic search in electronic databases, (ii) search of reference lists, and (iii) contacts to experts in the field.

We conducted a search covering 1 January 2000 to 30 November 2020 (stage one: 2002–2015, stage two: 2015–2020) using the databases PubMed, Web of Science, and PsycINFO (search strings in e-Appendix 2). In addition, we manually searched the reference lists of all included reviews and overview reviews. Based on the stage one search, we identified 72 preliminary eligible reviews and asked experts in the field to inform us of any potential additional eligible reviews that were not on the list. Experts included editors of 17 scientific journals and the presidents of five scientific organizations. A list of the experts is provided in e-Appendix 3.

### Study selection

After removing duplicates, we applied a four-step screening approach to exclude reviews not meeting the eligibility criteria. At each step, two reviewers conducted the screening independently from each other. Disagreements were solved by discussion. In case of uncertainty, the review in question was retained.

First, we screened all titles to determine whether the intervention was related to the workplace. Secondly, we screened the abstracts of the remaining reviews to determine whether the review included interventions aiming to improve the psychosocial work environment. Thirdly, we screened the full text of the remaining reviews to exclude reviews that did not match our inclusion and exclusion criteria. Finally, we did a full text reading of the remaining reviews to determine if all eligibility criteria were met.

### Quality assessment

Two researchers independently assessed the quality of the reviews using the “Health Evidence Quality Assessment Tool” (20), (e-Appendix 4). The instrument includes ten yes/no questions (table 1) yielding a total score of 0–10 points. Scores of 0–4, 5–7 and 8–10 points indicate weak, moderate, and strong quality, respectively. Disagreements were solved through discussion and by involvement of a third reviewer, if necessary.

**Table 1:** Quality assessment of the 76 reviews with strong, moderate or weak quality.

Quality assessment question	Reviews with strong, moderate or weak quality					
	Reviews (N) with ”yes” among the 20 reviews of strong quality	Average across the 20 reviews of strong quality	Reviews (N) with ”yes” among the 32 reviews of moderate quality	Average across the 32 reviews of moderate quality	Reviews (N) with ”yes” among the 24 reviews of weak quality	Average across the 24 reviews of weak quality
1. Are the population, intervention, and outcomes clearly described in the research question or inclusion criteria?	19	0.95	28	0.88	21	0.88
2. Were appropriate inclusion criteria used to select primary studies?	19	0.95	31	0.97	15	0.63
3. Did the authors describe a search strategy that was comprehensive?	10	0.50	12	0.38	6	0.25
4. Did the search strategy cover an adequate number of years?	20	1	31	0.97	20	0.83
5. Did the authors describe the level of evidence in the primary studies included in the review?	20	1	31	0.97	13	0.54
6. Did the review assess the methodological quality of the primary studies?	19	0.95	8	0.25	1	0.04
7. Are the quality of the primary studies assessed by a minimum of two authors and the method of conflict resolution described?	13	0.65	9	0.28	0	0
8. Was it appropriate to combine the findings of results across studies?	20	1	28	0.85	5	0.21
9. Were appropriate methods used for combining or comparing results across studies?	16	0.80	14	0.44	0	0
10. Do the data support the author’s interpretation?	20	1	8	0.25	0	0
Total quality score	176	8.80	200	6.25	82	3.41

### Data extraction

From each eligible review, we extracted name of first author, publication year, whether the review included a meta-analysis, number of primary studies, number of primary studies relevant for this overview of reviews, and the proportion of studies with a control group. We further recorded the investigated outcomes, the participants’ job groups, the type of interventions, and the main results of the review with regard to the organizational-level interventions. One researcher carried out the extraction, which was was checked by another researcher. Disagreements were solved through discussions and involvement of a third reviewer if necessary (see e-Appendix 5 for details).

Due to high heterogeneity in the interventions and outcomes, it was not feasible to conduct a meta-analysis.

Assessment of the effectiveness and the quality of evidence of the interventions

To judge the effectiveness of the interventions, BA and SJ independently assessed each review and summarized the main findings for outcomes of organizational-level interventions. Disagreements were resolved through discussions. We assessed the quality of evidence based on a rating system that we developed for this study, inspired by the rating systems used by Joyce et al (17) and the Navigation Guide (21). The rating system consists of five categories: strong, moderate, or low quality of evidence; inconclusive evidence due to lack of studies; and inconclusive evidence due to contradictory evidence. The definitions used for each of these categories are provided in e-Appendix 7. In short, our rating system, which was based on reviews as the unit of assessment, considered three aspects that we were able to retrieve from all reviews: The quality of the review (only reviews of high or moderate quality were considered), the consistency of results from multiple reviews (consistent versus less consistent results), and the proportion of controlled studies in the reviews (high, medium or low).

## Results

### Number of identified reviews

**Figure 2:**
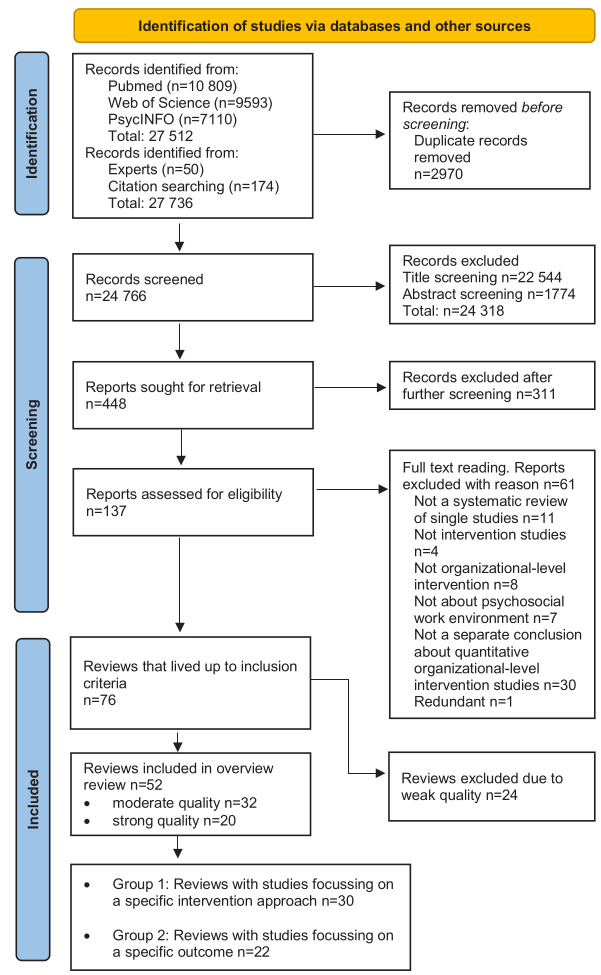
Flow chart of study selection for the meta-review.

Figure 2 shows the PRISMA flow diagram of the literature search and the selection of the studies. The electronic searches provided 27 512 records, the expert contacts 50 records, and the reference list search 174 records. Together 27 736 records were reduced to 24 766 records after removal of duplicates. After title and abstract screening, 448 records remained for a full text screening. Of those, 311 records were excluded because they did not match one or more of the inclusion criteria. The remaining 137 records were scrutinized in a round of detailed full text reading revealing that 61 further records were not eligible. A reference list of these articles with the reasons for exclusion is provided in e-Appendix 8. Seventy-six reviews met the criteria and were included in the quality assessment.

### Results of the quality assessment

Table 1 shows the results of the quality assessment of the 76 eligible reviews (see e-Appendix 9 for details). We judged 20, 32, and 24 reviews to be of strong (8–10 points), moderate (5–7 points), and weak quality (0–4 points), respectively. We subsequently excluded reviews of weak quality, leaving 52 reviews for the synthesis. The main reasons for assessing reviews as weak quality were that they did not: assess the quality of the included studies, assess the quality by at least two authors, appropriately combine results across studies, use appropriate methods for combining or comparing results across studies, or make interpretations supported by the data.

Most of the moderate quality reviews also had weaknesses in criteria questions 6–10 (table 1) but to a lesser degree than the weak quality reviews.

### Characteristics of included reviews and their primary studies

The 52 included reviews (6–9, 22–69) covered 957 primary studies: 30 focused on specific job groups or occupational sectors; 28 on the health care sector, 1 on school teachers, and 1 on male industrial workers. The remaining 22 reviews included intervention studies from various job groups. Only a minority of the included reviews (10 out of 52) conducted a meta-analysis.

The number of studies that overlapped between reviews in a category was in general low. Overlap of studies was highest in reviews about burnout (19 of 125 studies were reported in more than one review) and about introduction programs for newly trained nurses (11 of 127 studies were reported in more than one review) (see e-Appendices 5 and 6 for details). In most reviews, the proportion of studies with a control group [including randomized controlled trials (RCT)] was high (>50%) or medium (25–50%), while only 7 reviews had <25% of studies with a control group (e-Appendix 5).

For the analysis and synthesis, we divided reviews into two main groups based on their scope. Group 1 consisted of reviews investigating the effects of organizational-level interventions with specific organizational-level interventions (eg, what are the effects of interventions that aim to increase employees’ control?). Group 2 consisted of reviews investigating the effects of organizational-level interventions with a declared focus on changing employees’ health and wellbeing, and/or workplace retention (eg, what are the effects of interventions that aim to reduce burnout?).

### Synthesis of reviews that examined specific organizational-level interventions (group 1)

Table 2 shows the synthesis of the seven different intervention types (from 30 reviews) with a specific aim or approach. We found strong quality evidence for interventions on “changes in working time arrangements”, moderate quality evidence for interventions on “influence on work tasks or work organization”, “health care approach changes”, and “improvements of the psychosocial work environment”, and low quality evidence for interventions on “introduction programs for newly trained nurses” and for “prevention of workplace violence”. There was inconclusive evidence due to contradictory results for “leadership training or development” (e-Appendix 5).

**Table 2:** Synthesis on the quality of evidence of specific organizational-level interventions from 30 reviews (group 1)

Type of intervention	N reviews (R), studies (S), studies with a control group (CG)	Job groups	Quality of reviews	Quality of evidence	Comment ^a^	References
Changes in working time arrangements	4 R 76 S (38 CG)	Various	1 strong, 3 moderate	⋆⋆⋆ Strong	There is strong quality evidence that increasing workers’ influence on working time is effective for improving work-life balance. The intervention might also be effective with regard to health outcomes, but results are less consistent.	Joyce et al 2010 (27) Nijp et al 2012 (62) Bambra et al 2008 (45) Bambra et al 2008 (46)
Influence on work tasks or work organization	4 R 51 S (27 CG)	Various	1 strong, 3 moderate	⋆⋆ Moderate	There is moderate quality evidence for interventions that increase employee control can lead to positive health effects for employees. However, not all studies found positive results, which partly might be due to incomplete implementation. Interventions that were conducted for economic reasons seem to have a tendency for negative health effects.	Van Laethem et al 2013 (37) Aust et al 2004 (42) Bambra et al 2007 (47) Egan et al 2007 (55)
Health care approach changes	3 R 32 S (25 CG)	Health care staff	1 strong, 2 moderate	⋆⋆ Moderate	There is moderate quality evidence that interventions introducing health care approach changes can lead to improvements in employees’ knowledge, burnout, stress or job satisfaction.	Barbosa et al 2014 (48) Elliot et al 2012 (56) Spector et al 2016 (35)
Improvement of the psychosocial work environment	3 R 32 S (18 CG)	Nurses/ Various	1 strong, 2 moderate	⋆⋆ Moderate	There is moderate quality evidence for interventions that improve various aspects of the psychosocial work environment can lead to positive effects in the psychosocial work environment or employee wellbeing. Positive outcomes were found in studies that introduced workgroup activities that focused on better communication and support and in studies using a participative approach to enhance process aspects in the work environment and the core task.	Schalk et al 2010 (34) Daniels et al 2017 (53) Paguio et al 2019 (63)
Introduction programs for newly trained nurses	6 R 127 S (44 CG)	Nurses	2 strong, 4 moderate	⋆ Low	There is low quality evidence that introducing newly educated nurses to their first job through mentoring programs or other forms of systematic and supportive introduction show consistent results that it improves competencies. The intervention might also be effective with regard to decreasing turnover rate and improving job satisfaction, but results are less consistent.	Chen et al 2014 (50) Bakker et al 2020 (22) Brook et al 2019 (49) Zhang et al 2016 (69) Edwards et al 2015 (54) Missen et al 2014 (30)
Prevention of workplace violence	4 R 68 S (27 CG)	Health care staff	1 strong, 3 moderate	⋆ Low for effects on violence	There is low quality evidence that workplace violence prevention interventions can decrease violence.	Price et al 2015 (32) Anderson et al 2010 (41) Tölli et al 2017 (66) Kynoch et al 2011 (59)
Leadership training or development	6 R 310 S (182 CG)	Various / Health care staff	3 strong, 3 moderate	Inconclusive (contradictory results)	There is contradictory evidence if interventions about leadership trainings and development can lead to positive health and work environment effects for employees.	Collins et al 2004 (51) Grover et al 2016 (58) Gayed et al 2018 (25) Stuber et al 2021 (36) Kuehnl et al 2019 (28) Avolio et al 2009 (43)

^a^ For a detailed description of each review for the seven types of intervention, see Appendix 5, e-Table 5.1.

### Changes in working time arrangement (⋆⋆⋆ Strong quality evidence)

One review of strong quality (27) and three reviews of moderate quality (45, 46, 62) investigated the effects of changes in working time arrangements. All four reviews concluded that changes in working time arrangements, especially those that give employees more influence on scheduling their working time (eg, shifts), have positive effects on work environment outcomes (especially work-life balance). Influence on working time arrangements might also have positive health effects for employees; however, results were less consistent.

### Influence on work tasks or work organization (⋆⋆ Moderate quality evidence)

One review of strong quality (37) and three reviews of moderate quality (42, 47, 55) investigated the effects of increasing workers’ influence on work tasks or work organization. There was moderate quality evidence that increased control can lead to positive health effects. There was a tendency that the interventions were more likely to have a positive effect when the interventions were motivated by improving workers’ wellbeing compared to interventions that were motivated by improving the economy of the company.

### Health care approach changes (⋆⋆ Moderate quality evidence)

One review of strong quality (35) and two reviews of moderate quality (48, 56) examined the effects of introducing new approaches to dementia care on outcomes related to psychosocial work environment, health, and labor market affiliation of healthcare workers. All three reviews found some positive results for knowledge, burnout, stress, or job satisfaction; however, studies demonstrating null-effects or an absence of lasting effects were also found.

### Improvement of the psychosocial work environment (⋆⋆ Moderate quality evidence)

One review of strong (34) and two of moderate quality (53, 63) addressed organizational-level interventions aimed at improving the psychosocial work environment in general. There was moderate quality evidence that these types of interventions have positive effects on the psychosocial work environment or employee wellbeing. Positive outcomes were found in studies that introduced workgroup activities focusing on better communication and support and those using a participative approach to enhance procedural aspects in the work environment and the core task.

### Introduction program for newly trained nurses (⋆ Low quality evidence)

Two reviews of strong quality (22, 30) and four reviews of moderate quality (49, 50, 54, 69) investigated the effects of introduction programs, including mentor programs, for newly qualified nurses on various outcomes. There was low quality evidence for positive effects for improvement of competencies and inconsistent results for other outcomes, such as job satisfaction and turnover rates.

### Prevention of workplace violence (⋆ Low quality evidence)

One review of strong quality (32) and three reviews of moderate quality (41, 59, 66) examined the effects of interventions aimed at preventing violence from patients, which mostly included staff training. Three reviews (32, 59, 66) found that staff training can improve confidence, while two (32, 59) found that it can also increase knowledge. However, with regard to actually decreasing violence, we only found low quality evidence. One review found less clear results for violence reduction compared to other outcomes (32) while another review found clearer results for violence reduction compared to other outcomes (66).

### Leadership training or development (inconclusive evidence due to contradictory results)

Three reviews of strong quality (25, 28, 36) and three reviews of moderate quality (43, 51, 58) examined the effects of interventions aimed at leaders (eg, change in management approach or management development, coaching, and training). The evidence across reviews was different for leaders and for employees. Several reviews (25, 36, 43, 51) found positive effects for leaders, especially regarding their knowledge. However, the findings were contradictory with regard to employees: One review did not find effects on psychological symptoms for employees (25). Four reviews found mixed results for employee mental health (36) or wellbeing (28), moderate effects for organizational aspects (eg, employee job satisfaction) (51), and varying effects for employee work environment or health outcomes (58).

### Synthesis of reviews that examined interventions with a focus on changing employees’ health and wellbeing and workplace retention (group 2)

Table 3 shows the synthesis of the interventions on four specific outcomes (from 22 reviews). We found strong quality evidence for interventions about “burnout”, moderate quality evidence for interventions about “various health and wellbeing outcomes”, inconclusive evidence due to contradictory results for interventions about “stress” and inconclusive evidence due to lack of studies for interventions about “retention at work”.

**Table 3:** Synthesis of the quality of evidence of organizational-level interventions with a focus on employees’ health, wellbeing or labor market retention from 22 reviews (group 2).

Type of outcome	N reviews (R), studies (S), studies with a control group (CG)	Job groups	Quality of reviews	Quality of evidence	Comment ^a^	References
Burnout	8 R 125 S (38 CG)	Various / Health care staff	3 strong, 5 moderate	⋆⋆⋆ Strong	There is strong quality evidence that organizational-level interventions either by themselves or in combination with individual intervention components can reduce burnout.	Panagioti et al 2017 (64) Awa et al 2010 (44) Dreison et al 2018 (24) Pijpker et al 2020 (65) DeChant et al 2019 (9) Xu et al 2020 (40) West et al 2016 (39) Williams et al 2018 (68)
Various health and wellbeing outcomes	6 R 83 S (74 CG)	Various	3 strong, 3 moderate	⋆⋆ Moderate	There is moderate quality evidence that organizational-level interventions that aim to improve employees’ various health and wellbeing outcomes can lead to positive effects.	Montano et al 2014 (8) Corbiere et al 2009 (52) Gilbody et al 2006 (26) Romppanen et al 2016 (33) Lee et al 2014 (61) van Wyk et al 2010 (38)
Stress	6 R 47 S (43 CG)	Various	3 strong, 3 moderate	Inconclusive evidence due to contradictory results	There is contradictory evidence about the ability of organizational-level interventions to reduce stress.	Richardson et al 2008 (6) van der Klink et al 2001 (67) Giga et al 2003 (57) Ruotsalainen et al 2015 (7) Naghieh et al 2015 (31) Mimura et al 2003 (29)
Retention at work	2 R 6 S (1 CG)	Various / Health care staff	1 strong, 1 moderate	Inconclusive evidence due to lack of studies	There is inconclusive evidence due to lack of studies about the effect of organizational-level interventions on employee retention.	Cloostermans et al 2015 (23) Lartey et al 2014 (60)

^a^ For a detailed description of each review on the four outcomes, see e-Appendix 5, e-Table 5.2.

### Burnout (⋆⋆⋆ Strong quality evidence)

Three reviews of strong quality (24, 39, 40) and five reviews of moderate quality (9, 44, 64, 65, 68) examined burnout. Three reviews (24, 39, 64) conducted meta-analysis of organizational-level interventions and found consistent, albeit small, reductions in burnout scores. This was also true for most of the systematic reviews with a narrative synthesis (9, 65, 68), however, two reviews (40, 44) found also studies with mixed, no, or negative effects. Two reviews (24, 64) investigated if organizational-directed interventions are more effective than individual-directed interventions in reducing burnout but came to different conclusions favoring either organizational interventions (64) or individual interventions (24). One review (44) found that combined interventions show more positive and longer lasting results than exclusively organizational-level interventions and one review (65) examined combined interventions only and found positive effects. Overall, we assessed that there is strong quality evidence that organizational-level interventions either by themselves or in combination with individual intervention components can reduce burnout.

### Various health and wellbeing outcomes (⋆⋆ Moderate evidence)

Three reviews of strong quality (26, 33, 38) and three reviews of moderate quality (8, 52, 61) examined the effects of organizational-level interventions on various health and wellbeing outcomes. All reviews found that at least half of the intervention studies showed some positive effects. In a few cases, the positive effects were only found for high-risk employees or those that received a high dose of the intervention (33, 61). Two of the six reviews found a tendency for better effects from combined individual and organizational-level interventions (52) or from more comprehensive interventions tackling many organizational aspects at once (8). Overall, we assessed moderate quality evidence that organizational-level interventions that aim to improve employees’ various health and wellbeing outcomes can lead to positive effects.

### Stress (inconclusive evidence due to contradictory results)

Three reviews of strong quality (7, 29, 31) and three reviews of moderate quality (6, 57, 67) examined the effects of organizational-level interventions on stress and came to different conclusions. Two reviews (6, 67) found no effects of organizational stress interventions based on meta-analysis with five studies each. One review (7) concluded that the only organizational-level interventions that showed positive effects were those that improved working time schedules. The other three reviews came to more positive conclusions. One review (57) found some positive effects in all types of interventions, including organizational-level interventions, and pointed out that individual-level interventions were less likely to result in longer lasting effects than organizational-level interventions. Another review (31) found weak evidence, however all studies in this review also included individual-level intervention components. And one review (29) found potentials for positive effects, but could not conclude due to too few studies. Overall, we assessed that there is inconclusive evidence about the ability of organizational-level interventions to reduce stress.

### Retention at work (inconclusive evidence due to lack of studies)

One review of strong quality that included various job groups (23) and one of moderate quality that included nurses (60) investigated interventions to improve retention of older employees. Although all six identified studies showed some positive effects on retention, neither review reached a conclusion because there were too few studies. Both pointed out that interventions initiating multiple strategies at the same time may be better suited for the retention of older employees.

## Discussion

### Summary of evidence

In this overview of reviews, we identified 52 reviews covering 957 primary studies of organizational-level interventions across a variety of job groups. We found strong or moderate quality of evidence for four specific intervention approaches and two work environment outcomes. This suggests that several types of organizational-level interventions can lead to improvements for employees.

Among the 30 reviews studying specific organizational-level interventions, “changes in working time arrangements” was the only intervention approach with a strong quality of evidence, especially with regard to interventions that increased workers’ influence on working time. The direct link between the content of the intervention in many of these studies (eg, more flexibility) and the measured outcome (eg, work-life balance) might have played a role for the effectiveness of this type of intervention. In addition, working time changes are typically implemented centrally for the entire workplace, thereby guaranteeing a high implementation degree, which might have contributed to the positive effects. Most other organizational-level workplace interventions of the psychosocial work environment require more complex and time-consuming implementation activities, where employees and their leaders need to agree on changes and implement them so that enough employees are exposed to them (70), which can lead to less consistent results across studies. We found moderate quality evidence for positive effects of the three intervention types “influence on work tasks or work organization”, “health care approach changes”, and “improvements of the psychosocial work environment”, and low quality evidence for interventions focusing on “introduction programs for newly trained nurses” and for “prevention of workplace violence”.

The results suggest that it might be easier to reach the more proximal effects of the interventions (improvements in the psychosocial work environment) than it is to reach the more distal effects (health and retention) (see figure 1). In this group of reviews, distal effects for health outcomes were only found for the two intervention types “influence on work tasks or work organization” and “health care approach changes”, while none of the interventions types in this group found effects for retention.

Regarding “leadership training or development”, we found inconclusive evidence due to contradictory results. Although several reviews reported positive effects for leaders, the results for employees varied considerably. Again, it may be easier to reach effects that are closer to the content of intervention (teaching knowledge about leadership styles) than to change the more distal outcomes (improvement in employee outcomes due to changes in leadership style).

Among the 22 reviews that examined organizational-level interventions with a focus on specific outcomes, only interventions aiming to reduce burnout had high quality of evidence, whereas interventions aiming to improve employees’ various health and wellbeing outcomes had moderate quality of evidence. Evidence was inconclusive for interventions aiming to improve retention (due to lack of studies) and for interventions aiming to reduce stress (due to contradictory results).

Of the eight reviews focusing on burnout, several included interventions that covered a wide variety of organizational-level changes (job training, teamwork, workflow changes etc.). One review (65) performed a moderator analysis which suggested that enhanced job control, social support, and elimination of stressors explain the effectiveness of the interventions. The findings of the other reviews also pointed at these aspects highlighting that especially interventions focusing on work schedules, workload reductions, improved work organization, enhanced job-control and participation, social support, communication, feedback, supervision and leadership support lead to positive effects. The high quality of evidence of these interventions might be due to that the interventions were able to cause substantial changes in the work environment (proximal effects), which, in turn, led to changes in burnout (distal effect). The combination with individual-level interventions in many burnout interventions might have further contributed to the positive effects. It needs to be noted, though, that the effect sizes of the burnout interventions were small. This was partly expected as a considerable part of the interventions were conducted with healthy employees. As Tanner-Smith et al (71) have pointed out, the magnitude of intervention effect sizes should be evaluated relative to the context of the intervention area and there is no clear rule to determine when an effect size is too small or large enough. Therefore, we did not consider effect size in the quality of evidence assessment.

The conclusions of the reviews investigating interventions to reduce stress were contradictory. The three systematic reviews that reported no effects (6, 67) or low quality evidence for one specific type of interventions (improved working time schedules) (7) all conducted meta-analyses, while the other three systematic reviews synthetized the results narratively (29, 31, 57). The meta-analyses included studies both with and without effects, and did not show an effect after pooling the results and taking sample size into consideration. Compared to that, the reviews with narrative synthesis reported a variety of effects found in each study without taking sample size into account and concluded “some” evidence (57), “weak” evidence (31) or “tendencies” (29).

### Interpretation of the results in the context of other overviews of reviews

Our findings show similarities but also differences to previous overviews of reviews. Corresponding with our findings, the overview of reviews by Bambra et al (13) and Joyce et al (17) reported that increasing employee involvement (in general or specifically regarding control on working hours) overall has positive effects on employee health and wellbeing. Bhui et al (14), on the other hand, assessed mixed evidence for organizational-level interventions. Williams-Whitt et al (16) reported positive results for absenteeism, productivity and cost-effectivity for interventions that reduced job demands and increase job control. The reason for their more positive assessment of organizational-level interventions might have been that they included many reviews that investigated the reduction of physical work demands, whereas we focused on psychosocial work environment factors only. Similarly, the conclusion of the overview of reviews by Wagner et al (15) was more positive than ours as it reported mental health interventions to have positive impacts on workplace outcomes (e.g. absenteeism). Wagner et al included reviews focusing on individual interventions as well as interventions consisting of secondary and tertiary preventive measures, whereas our overview review focused mainly on primary prevention interventions conducted in healthy populations.

### Strengths and limitations of the overview of reviews

This article provides the most comprehensive overview of knowledge on the effectiveness of organizational-level psychosocial work environment interventions to date. Compared with other overviews of reviews of interventions targeting the psychosocial work environment (13–17), our study identified the largest number of reviews by far, covering almost one thousand organizational-level intervention studies. The identification of 52 reviews of moderate or strong quality made it possible to investigate the quality of evidence of different types of organizational-level interventions. We showed that organizational-level interventions can differ in their complexity regarding implementation, and that only a few types of interventions with moderate-to-strong quality of evidence have been demonstrated to make changes in the more distal health outcomes.

We cannot rule out that we have overlooked relevant reviews, although we consider the likelihood low because of our comprehensive search strategy. Publication bias is a concern because primary intervention studies with null findings or negative effects of interventions might be underrepresented in the literature as authors and editors may tend to be less interested to publish these results, despite their potential for learning (72). Publication bias would likely cause an overestimation of the positive effects of the interventions.

We checked if reviews investigating the same type of interventions were based on the same primary studies and found that this only happened to a low degree (for details see e-Appendices 5 and 6). However, this also shows that even reviews that aim to study the same topic often differ in their inclusion and exclusion criteria, leading to the identification of different studies and potentially different conclusions. The overview review approach allowed us to make these inconsistencies visible and to ensure the most comprehensive overview of organizational-level interventions.

Most reviews had a proportion of controlled studies above 50%, but the number of studies using RCT design was low in most reviews (e-Appendix 5). Although RCT are often considered to produce the most reliable evidence, there is also doubt if this is the most appropriate approach to organizational-level intervention studies that require flexibility in order to fit the specific workplace context, in which they are implemented (73–76). For our assessment of the quality of evidence, we therefore chose to use the proportion of controlled studies, rather than proportion of RCT, as one of the three aspects for the assessment (for details see e-Appendix 7).

Only 10 of 52 reviews conducted a meta-analysis, while the majority of reviews argued that the interventions were too diverse to pool results for statistical analysis. This might have contributed to more positive conclusions. As can be seen in the divergent conclusions of reviews investigating interventions to reduce stress, reviews with narrative syntheses typically do not weigh population size when comparing studies, which increases the likelihood that studies with smaller populations contribute more to the overall assessment than they would in a meta-analysis. In contrast, meta-analyses are often focused on only one particular outcome and might therefore miss other outcomes thereby potentially underestimating the effectiveness of interventions.

Our rating system for the assessment of the effectiveness and the quality of evidence of the interventions was rather broad. As our “unit of assessment” were systematic reviews, we could not use traditional rating systems that assess the quality of primary studies. We were inspired by the Navigation Guide with regard to the general assessment of the overall level of evidence and by a rating system used in another similar overview review (17). As shown in e-Appendix 7, our rating system was based on three criteria that we were able to retrieve from all reviews: The quality of the review (which we assessed by using the “Health Evidence Quality Assessment Tool”, an established instrument), the consistency of results from multiple reviews, and the proportion of controlled studies in the reviews. With this rating system we aimed to provide a “user friendly” summary of the breadth of research, a central aim of overview reviews (12). We acknowledge that other rating systems, eg, rating systems with a stronger focus on RCT, could have reached other conclusions than we did.

We categorized the reviews into groups of reviews with similar aims. This categorization was not a problem with regard to specific organizational-level interventions; however, it was more challenging with regard to the reviews focusing on specific outcomes, especially the three health and wellbeing outcomes. We carefully checked the aims in these reviews and found that some reviews were more focused on a specific outcome, while others had a broader approach. All eight reviews that investigated burnout had searched specifically for primary studies that measured burnout with a validated tool. All reviews in the group that investigated stress had searched for primary studies about stress-management or stress reduction. The reviews that we grouped under the label “various health and wellbeing outcomes” had a broader approach and included primary studies that measured for example depressive symptoms, wellbeing, work-life balance, job satisfaction, absenteeism, staff turnover or job performance. Some of the primary studies also measured burnout or stress, but since the search of the reviews was not restricted to these outcomes, they included a much broader variety of studies.

Some of the findings were in agreement across review groups. For example, the design of work schedules that either give employees more flexibility or other advantages, like less weekend shift, were found to not only be associated with improvement of work-life balance in the group of reviews investigating changes in working time arrangements, but were also found to be associated with a reduction of burnout and stress in the reviews that studied these outcomes. Employees’ influence and participation also played a central role in different types of interventions, including interventions on certain aspects of work (for example increasing influence on the work-schedules), but also through discussion groups that aimed to increase employees’ influence on the way work is organized (which included interventions on burnout). This aligns well both with the prominent role that autonomy (or job control) plays in the literature on work design and stress (77, 78) and with the results from a recent qualitative study that corroborated that influence is a key factor for mental health of contemporary employees in knowledge and relational work (79).

Most studies in the included reviews were conducted in Europe, North America, Australia, Japan, and a few other Asian countries, while experiences from other parts of the world were largely missing. In addition, 28 of the 52 reviews exclusively focused on interventions conducted in the health care sector. The results are therefore mostly representative for workplaces in high-income countries and in sectors with large organizations, while the number of workplace intervention studies in small and medium sized enterprises is only slowly increasing (80, 81). Experiences from other parts of the world and in sectors that have less favorable conditions for doing organizational interventions and/or research (eg, construction) seem to be much more difficult to gather systematically (82).

### Implications

Our systematic overview of reviews suggest that organizational-level interventions have the potential to improve both employee psychosocial work environment and health outcomes. That means that both adverse psychosocial working conditions and adverse health outcomes are potentially, at least partly, preventable through appropriate changes in the psychosocial work environment. However, not all interventions led to the expected improvements, and some studies even reported negative effects of the interventions. The success of organizational-level interventions may depend on certain conditions like sufficient and continuous management support, appropriate problem assessment so the intervention fits to the problems to be solved, and the active involvement of employees (77, 83–85). However, unforeseeable contextual changes such as restructuring, downsizing, high turnover among leaders and/or employees, competing projects, and similar incidents that disturb or limit the focus needed to develop and implement workplace improvements, may impact the chances for positive outcomes (86).

Although knowledge about implementation aspects is growing (87, 88), more research is needed to better understand why certain organizational-level interventions lead to the desired changes while others do not. Exclusively reviewing the effects of intervention studies will not provide this knowledge (74, 75). Instead, we need studies that systematically investigate if the necessary preconditions for the interventions were in place, which contextual aspects might have had an influence on the intervention (83, 88), and the extent to which failures can be attributed to implementation problems (26, 89). Future studies, reviews, and overviews of reviews therefore should systematically assess the implementation of the intervention before evaluating the effects (37). In addition, more systematic research on implementation processes will help identifying essential factors for successful organizational-level interventions (83, 90).

### Concluding remarks

In this overview of reviews on organizational-level interventions, we identified moderate or high quality of evidence for effectiveness for four intervention approaches and two outcomes. This suggests that the work environment and health outcomes of employees can be improved by certain organizational-level interventions. However, not all organizational-level interventions led to positive effects, and the evidence is low or inconclusive for several types of interventions. Implementation and context are increasingly being assessed in intervention studies, but this knowledge also needs to be synthesized in reviews and overviews of reviews to better understand what it takes for organizational-level intervention to be effective.

## Supplementary material

Supplementary material
