# Supplementary material for: How effective are organizational-level interventions in improving the psychosocial work environment, health, and retention of workers? A systematic overview of systematic reviews
Source: Scand J Work Environ Health. 2023 Jun 30;49(5):315–29. doi: 10.5271/sjweh.4097 (PMC10713994; doi:10.5271/sjweh.4097)
Supplement: Supplementary material [file SJWEH-49-315-S001.pdf]

How effective are organizational-level interventions in improving the psychosocial work environment, health, and retention of workers? A systematic overview of systematic reviews[1]

by Birgit Aust, PhD,[2] Jeppe Lykke Møller, PhD, Mads Nordentoft, PhD, Karen Bo Frydendall, PhD, Elizabeth Bengtsen, Andreas Brøgger Jensen, MSc, Anne Helene Garde, PhD, Michiel Kompier, PhD, Norbert Semmer, PhD, Reiner Rugulies, PhD, Sofie Østergaard Jaspers, PhD

1. *Supplementary material*
2. *Correspondence to: Birgit Aust, PhD, National Research Centre for the Working Environment, Lersø Parkallé 105, DK-2100 Copenhagen, Denmark. [E-mail: bma@nfa.dk]*

Table of contents:

	page
e-Appendix 1: The two stages of the overview of reviews	2
e-Appendix 2: Search strings	3-5
e-Appendix 3: List of experts	6
e-Appendix 4: Health Evidence Quality Assessment Tool	7
e-Appendix 5: Detailed description of the included reviews and synthesis	
e-table 5.1 Synthesis of the reviews in group 1	8-18
e-table 5.2 Synthesis of the reviews in group 2	19-28
e-Appendix 6: Overlap between primary studies in the three review groups about burnout, various health and wellbeing outcomes and stress.	29
e-table 6.1 Overlap of primary studies in reviews on burnout	29
e-table 6.2 Overlap of primary studies in reviews on various health and wellbeing outcomes	30
e-table 6.3 Overlap of primary studies in reviews on stress	30
e-Appendix 7: Levels of quality of evidence	31
e-table 7.1 Illustration of the levels of quality of evidence	32
e-Appendix 8: List of 61 excluded reviews with reason	33-34
e-Appendix 9: Quality assessment of the 76 reviews with strong, moderate or weak quality	35-37
References	38-43

e-Appendix 1: The two stages during which the overview of reviews was conducted

We conducted the overview of reviews in two stages. Stage one was completed in 2017, when we published a report of the overview of reviews in Danish language on the home page of the Danish National Research Centre for the Working Environment (1).

For stage one, we conducted a literature search covering the time span from 2000 to 2015. We identified 72 eligible reviews and we asked experts in the fields to inform us of any potential additional eligible article that was not on the list (see **e-appendix 3** below for further details on the experts). In addition, we searched manually the reference lists of all included reviews and of other key articles in the field.

We identified 47 reviews that matched our eligibility criteria at stage one. All reviews were assessed for quality and 16 reviews that were assessed to have weak quality were excluded. Thus, stage one ended with the identification of 31 reviews of moderate (21 reviews) or strong (10 reviews) quality.

At stage two, we prepared the present manuscript for the international research community with an updated literature search until November 2020. We updated our search in the electronic databases and our search in reference lists of newly identified reviews. We used exactly the same search strings as in the first round (see **e-Appendix 2** below) and searched the three databases from the date we ended in stage one until November 30th 2020. We identified several new reviews, resulting in a total of 52 eligible reviews for stage one and stage two combined, 32 of moderate and 20 of strong quality.

e-Appendix 2: Search strings

Search string PubMed

#1 (workplace marker)

employee*[Text Word]) OR workplace[MeSH Terms] OR job site*[Text Word]) OR (organization and administration[MeSH Terms]) OR organization*[Text Word] OR organisation*[Text Word] OR organizations[MeSH Terms] OR team[Text Word] OR teams[Text Word] OR "work location"[Text Word] OR "workplace"[Text Word] OR "work place"[Text Word] OR "work site"[Text Word] OR worker*[Text Word] OR *occupational[Text Word] OR work[Text Word]) OR workplace[MeSH Terms] OR workplace* OR work place* OR worksite* OR work site** **Filters: Journal Article; English**

AND

#2 (psychosocial marker)

autonomy[Title/Abstract]) OR communication[Text Word]) OR control[Title/Abstract]) OR demands[Text Word] OR demand[Text Word) OR empowerment[Text Word]) OR feedback[MeSH Terms]) OR feedback[Title/Abstract]) OR involve*[Title/Abstract]) OR "job stress"[Text Word]) OR "occupational stress"[Text Word]) OR participation[Text Word]) OR psychological[Title/Abstract]) OR psychosocial[All Fields] OR "role clarification"[Text Word]) OR "role conflict"[Text Word] OR "role conflicts"[Text Word] OR "role imbalance"[Text Word] OR "job security"[Title/Abstract]) OR "shift work"[Text Word] OR "shift worker"[Text Word] OR "shift workers"[Text Word]) OR social capital[MeSH Terms] OR "social capital"[Text Word] OR "social networks"[Text Word] OR "social network"[Text Word)) OR social support[MeSH Terms] OR "social relationships"[Text Word] "social relationship"[Text Word OR stress, psychological[MeSH Terms]) OR (stress[Title/Abstract] OR stressor*[Title/Abstract] OR stress, physiological[MeSH Terms] OR support[Title/Abstract] OR "task characteristics"[Text Word] OR "task structure"[Text Word] OR decision making[MeSH Terms] OR internal external control[MeSH Terms]) OR "team work"[Text Word] OR trust[MeSH Terms] OR trust[Title/Abstract] OR "working time"[Text Word] OR participative[Text Word] OR "flexible working conditions"[Text Word] OR collaborat*[Title/Abstract] OR "occupational health psychology"[Text Word] OR leader*[Text Word]) OR manager*[Text Word]) OR management[Title/Abstract]) OR "job strain"[Text Word]) OR ("effort reward imbalance" OR "effort-reward imbalance") OR bullying[MeSH Terms] OR bullying[Text Word]) OR ("organizational justice" OR "organisational justice") OR "job insecurity"[Text Word]) OR ("working hour"[Text Word] OR "working hours"[Text Word]) OR motivation[MeSH Terms] OR motivation[Text Word]) OR engagement[Title/Abstract]) OR job satisfaction[MeSH Terms] OR "job satisfaction"[Text Word]) OR discriminat*[Title/Abstract] OR "workplace democracy"[Text Word]) OR influenc*[Title/Abstract] OR ("organizational citizenship behavior" OR "organisational citizenship behaviour") OR ("organization citizenship behavior" OR "organisation citizenship behaviour") OR "team training"[Text Word] OR "team building"[Text Word] OR "job design"[Text Word]) OR "self management"[Title/Abstract] OR self manag*[Title/Abstract] OR conflict[MeSH Terms] OR conflict[Title/Abstract] OR well-being[Text Word] **Filters: Journal Article; English**

#1 AND #2 AND

#3 (interventions)

intervention studies[MeSH Terms] OR intervention*[Text Word]) OR "intervention study"[Text Word] OR "intervention studies"[Text Word] **Filters: Journal Article; English**
OR trial[Title/Abstract]) OR transformation*[Text Word] **Filters: Journal Article; English**

#1 AND #2 AND #3 AND

#4 (reviews)

"cochrane database syst rev"[Journal]) OR search*[Title/Abstract]) OR meta-analysis [Publication Type]) OR MEDLINE[Title/Abstract]) OR (systematic[Title/Abstract]) AND review[Title/Abstract]) **Filters: Journal Article; English**

#1 AND #2 AND #3 AND #4

Search string Web of Science

#1 (workplace marker)

TOPIC: (employee*) *OR* **TOPIC:** (workplace*) *OR* **TOPIC:** ("job site" OR "job sites") *OR* **TOPIC:** (organization* OR organisation*) *OR* **TOPIC:** (team*) *OR* **TOPIC:** (work location*) *OR* **TOPIC:** (workplace* OR work place*) *OR* **TOPIC:** (work site* OR worksite*) *OR* **TOPIC:** (worker*) *OR* **TOPIC:** (occupational OR work*) **Refined by: LANGUAGES:** (ENGLISH) AND **DOCUMENT TYPES:** (ARTICLE)

AND

#2 (psychosocial marker)

TOPIC: (autonomy) *OR* **TOPIC:** (communication) *OR* **TOPIC:** (control) *OR* **TOPIC:** (demands OR demand) *OR* **TOPIC:** (empowerment) *OR* **TOPIC:** (feedback) *OR* **TOPIC:** (involve*) *OR* **TOPIC:** ("job stress" OR "occupational stress") *OR* **TOPIC:** (participation) *OR* **TOPIC:** (psychological) *OR* **TOPIC:** (psychosocial) *OR* **TOPIC:** ("role clarification") *OR* **TOPIC:** ("role conflict" OR "role conflicts") OR **TOPIC**=("role imbalance") OR **TOPIC:** ("job security") *OR* **TOPIC:** ("shift work") *OR* **TOPIC:** ("shift worker" OR "shift workers") *OR* **TOPIC:** ("social capital") *OR* **TOPIC:** ("social network") OR **TOPIC**=("social networks") *OR* **TOPIC:** ("social support") OR **TOPIC:** ("social relationship" OR "social relationships") *OR* **TOPIC:** (stress OR stressor*) *OR* **TOPIC:** (support) *OR* **TOPIC:** ("task characteristics") *OR* **TOPIC:** ("task structure") *OR* **TOPIC:** ("team work") *OR* **TOPIC:** (trust) *OR* **TOPIC:** ("working time") *OR* **TOPIC:** (participative) *OR* **TOPIC:** ("flexible working conditions") *OR* **TOPIC:** (collaborat*) *OR* **TOPIC:** ("occupational health psychology") OR **TOPIC:** (leader*) *OR* **TOPIC:** (manager*) *OR* **TOPIC:** (management) *OR* **TOPIC:** ("job strain") *OR* **TOPIC:** ("effort reward imbalance") *OR* **TOPIC:** (bullying) *OR* **TOPIC:** ("organizational justice" OR "organisational justice") *OR* **TOPIC:** ("job insecurity") *OR* **TOPIC:** ("working hour" OR "working hours") *OR* **TOPIC:** (motivation OR "well-being") *OR* **TOPIC:** (engagement) *OR* **TOPIC:** ("job satisfaction") *OR* **TOPIC:** (discriminat*) *OR* **TOPIC:** ("workplace democracy") *OR* **TOPIC:** (influenc*) *OR* **TOPIC:** ("organizational citizenship behavior") *OR* **TOPIC:** ("organisational citizenship behaviour") *OR* **TOPIC:** ("organization citizenship behavior") *OR* **TOPIC:** ("organisation citizenship behaviour") *OR* **TOPIC:** ("team training") *OR* **TOPIC:** ("team building") *OR* **TOPIC:** ("job design") *OR* **TOPIC:** ("self-management") *OR* **TOPIC:** (self manag*) *OR* **TOPIC:** (conflict)
Refined by: LANGUAGES: (ENGLISH) AND **DOCUMENT TYPES:** (ARTICLE)

AND

#3 (interventions)

TOPIC: (intervention*) *OR* **TOPIC:** (trial) *OR* **TOPIC:** (transformation*)
Refined by: LANGUAGES: (ENGLISH) AND **DOCUMENT TYPES:** (ARTICLE)

AND

#4 (reviews)

TOPIC: (cochrane) *OR* **TOPIC:** (Medline OR Pubmed) *OR* **TOPIC:** (search*) *OR* **TOPIC:** ("systematic review") *OR* **TOPIC:** ("systematic reviews") *OR* **TOPIC:** (systematic review) *OR* **TOPIC:** ("meta analyses" OR "meta analysis") *OR* **TOPIC:** (meta-analys?s)
Refined by: LANGUAGES: (ENGLISH) AND **DOCUMENT TYPES:** (ARTICLE)

#1 AND #2 AND #3 AND #4

Search string PsycINFO

#1 (workplace marker)

employee*.mp. OR (job adj1 site*).mp. OR organi#ation*.mp. OR team*.mp. OR (work adj1 location*).mp. OR workplace*.mp. OR (work adj1 site*).mp. OR worksite*.mp. OR worker*.mp. OR occupation*.mp. OR work*.mp. AND **limit to (peer reviewed journal and English language**

AND

#2 (psychosocial marker)

autonomy.ti,ab. OR communication.mp. OR control.ti,ab. OR (demands OR demand).ti,ab. OR empowerment.mp. OR feedback.ti,ab. OR involve*.mp. OR (job adj1 stress).mp. OR (occupational adj1 stress).mp. OR participation.mp. OR psychological.ti,ab. OR psychosocial.mp. OR (role adj1 clarification) OR (role adj1 conflict) OR (role adj1 imbalance) OR (job adj1 security) OR shift work*.mp. OR (social adj1 capital) OR (social adj1 network*).mp.OR (social adj1 support).mp. OR (social adj1 relationship*) OR (stress OR stressor*).mp. OR support.ti,ab. OR leader*.mp. OR manager*.mp. OR management.ti,ab. OR (job adj1 strain).mp. OR (effort adj1 reward adj1 imbalance).mp. OR bullying.mp. OR (organi#ational adj1 justice).mp. OR (job adj1 insecurity).mp. OR (working adj1 hours).mp. OR motivation.mp. OR engagement.ti,ab. OR (job adj1 satisfaction).mp. OR discriminat*.ti,ab. OR (workplace adj1 democracy).mp. OR influenc*.mp. OR (task adj1 characteristics).mp. OR (task adj1 structure).mp. **OR (team adj1 work).mp. OR trust.mp. OR (working adj1 time).mp. OR participative.ti,ab. OR (flexible adj1 working adj1 conditions).mp. OR collaborat*.mp. OR (occupational adj1 health adj1 psychology).mp. OR (organi#ational adj1 citizenship adj1 behavior?).mp. OR (team adj1 training).mp. OR (team adj1 building).mp** AND **limit to (peer reviewed journal and English language**

#1 AND #2

AND

#3 (interventions)

Intervention*mp. OR trial.ti,ab. OR transformation*.mp. AND **limit to (peer reviewed journal and English language**

#1 AND #2 AND #3

AND

#4 (reviews)

Cochrane.ti,ab,jn. OR MEDLINE.mp. OR pubmed.mp. OR search* OR (systematic adj1 review*).mp.OR (systematic adj1 review*).mp. OR (meta adj1 analys#s).mp. OR review.ti,ab. AND **limit to (peer reviewed journal and English language**

#1 AND #2 AND #3 AND #4

e-Appendix 3: List of experts

Journal editors for:

- Work & Stress
- Scandinavian Journal of Work Environment and Health
- Occupational and Environmental Medicine
- Social Science and Medicine
- Journal of Occupational and Organizational Psychology
- Journal of Occupational Health Psychology
- European Journal of Work and Organizational Psychology
- International Journal of Stress Management
- WORK
- Stress & Health
- International Journal of Occupational and Environmental Health
- International Archives of Occupational and Environmental Health
- American Journal of Industrial Medicine
- Annals of Occupational Hygiene
- Occupational Medicine
- Journal of Occupational Health
- Stress

Organisations:

- International Commission on Occupational Health. Scientific Committee on Work Organisation and Psychosocial Factors (ICOH-WOPS)
- The European Public Health Association (EUPHA), Section on Social Security, Work and Health
- European Academy of Occupational Health Psychology (EAOHP)
- Scientific Committee on Epidemiology in Occupational Health (EPICOH)
- Conference on the Prevention of Work-Related Musculoskeletal Disorders (PREMUS)

All experts were contacted in stage one only. In addition to the journal editors and the organizations, we also contacted two international experts (Professor Michiel Kompier, Radboud University, The Netherlands and Professor Emeritus Norbert Semmer, University of Bern, Switzerland) and three experts at the National Research Centre for the Working Environment, Copenhagen, Denmark (Dr. Vilhelm Borg, Professor Anne Helene Garde, and Professor Reiner Rugulies). After we completed stage two, Professors Kompier, Semmer, Garde and Rugulies contributed to the interpretation of the results and the writing of the present paper and were included as co-authors.

e-Appendix 4: Health Evidence Quality Assessment Tool

First Author: ____________________ Journal: ____________________

Year: ____________________ Reviewer: ____________________

CRITERION	Yes	No
**Q1**. Are the population, intervention, and outcomes clearly described in the research question or inclusion criteria?		
**Q2**: Were appropriate inclusion criteria used to select primary studies?		
**Q3:** Did the authors describe a search strategy that was comprehensive?<br>(at least two strategies from each column)<br>*Circle all strategies used*<br>• Health databases<br>• Psychological databases<br>• Social science databases<br>• Educational databases<br>• Other<br>• Handsearching<br>• Key informants<br>• Reference lists<br>• Unpublished		
**Q4:** Did the search strategy cover an adequate number of years?		
**Q5:** Did the authors describe the level of evidence in the primary studies included in the review?<br>*Circle the appropriate level of evidence*<br>• Level I → RCTs only<br>• Level II → Non-randomized, cohort, case-control<br>• Level III → Uncontrolled studies		
**Q6:** Did the review assess the methodological quality of the primary studies?<br>Place a check mark in the Yes column if the following **three** criteria are met:<br>1. Each primary study should be assessed for methodological quality using a standardized assessment tool/scale.<br>2. A minimum of four out of the following seven areas should be assessed and the results described for each included primary study (the first three areas needed to be fulfilled):<br>• **Research design**<br>• **Data collection methods**<br>• **Follow-up rates / attrition rates**<br>• Study sample<br>• Participation rates<br>• Data analysis<br>• Sources of bias<br>3. The implication of the quality-related data on the review's findings must be addressed		
**Q7:** Are the quality of the primary studies assessed by a minimum of two authors and the method of conflict resolution described? **OR:** Are an inter-rater agreement Kappa score of at least 0.80 reported?		
**Q8:** Was it appropriate to combine the findings of results across studies?		
**Q9:** Were appropriate methods used for combining or comparing results across studies?		
**Q10:** Do the data support the author's interpretation?		
TOTAL SCORE		

Quality Assessment Rating: **Strong:** 8-10 **Moderate:** 5-7 **Weak:** 4 or less

e-Appendix 5: Detailed description of the included reviews and synthesis

e-Table 5.1: Synthesis of the 30 reviews that examined specific organizational-level interventions (group 1)

1. Changes in working time arrangements (4 reviews/76 studies)					
**Reference (Job groups)**	**Quality of review**	**Type of interventions (Number of studies relevant for this overview) and study designs of relevant studies**	**Outcomes**	**Main results (Meta-analysis: Yes/No)**	**Proportion of studies with a control group** High: More than 50% studies with a control group Medium: 50% - 25% Low: Less than 25%
Joyce et al 2010 (2) (Various job groups)	Strong quality review	Flexible working conditions, including flexible working time arrangements, flexible contracts with regard to part-time retirement, and part-time work (10 out of 10 studies): 10 controlled before and after studies	Various health outcomes and wellbeing	Effective regarding health outcomes for interventions that increase control of working time (such as self-scheduling) and choice (such as gradual/partial retirement) (Meta-analysis: No)	High
Nijp et al 2012 (3) (Various job groups)	Moderate quality review	Working time arrangements (5 out of 53 studies): 5 controlled before and after studies	Work-life balance, various health outcomes, job-related outcomes such as job satisfaction and turnover	Effective regarding work-life balance. Insufficient evidence for job related outcomes. No overall effect for health/wellbeing. (Meta-analysis: No)	High
Bambra et al 2008 (4) (Various job groups)	Moderate quality review	Working time arrangements among shift workers (21 studies published in 26 articles): 1 crossover–controlled trial, 12 controlled prospective cohort studies, 8 uncontrolled prospective cohort studies	Work-life balance, various health outcomes	Effective regarding health and work-life balance for forward and for fast shift rotation and for self-scheduling of shift. (Meta-analysis: No)	High

Bambra et al 2008 (5) (Various job groups)	Moderate quality review	Comprised working weeks among shift workers, i.e. working longer hours on each day but working fewer days per week (40 out of 40 studies): 5 controlled prospective cohorts, 18 uncontrolled prospective cohorts. 3 prospective repeat cross-section studies with control group, 2 prospective repeat cross-section studies, 2 retrospective cohort studies with control group, 9 retrospective cohort studies, 1 retrospective repeat cross-section study	Work-life balance, various health outcomes	Effective regarding work-life balance. Inconsistent results regarding health outcomes.<br>(Meta-analysis: No)	Medium
**Conclusion**<br>Together, the four reviews in this section covered 76 studies (3 studies are included in more than one review). Half of the studies had a control group (38), 0 RCTs.<br><br>⋆⋆⋆ **Strong quality of evidence:** Consistent results from multiple reviews with high or moderate quality and with a high proportion of studies with a control group.<br>Specifically: Three reviews (Joyce et al 2010 (2), Nijp et al 2012 (3)**,** Bambra et al 2008 (4)) with a high proportion of studies with a control group found positive results for work-life balance.<br><br>**There is strong quality of evidence that increasing workers' influence on working time is effective for improving work-life balance. The intervention might also be effective with regard to health outcomes, but results are less consistent.**					

**2. Influence on work tasks or work organization (4 reviews/51 studies)**					
**Reference (Job groups)**	**Quality of review**	**Type of Intervention (number of studies relevant for this overview) and study designs of relevant studies**	**Outcomes**	**Main results (Meta-analysis: Yes/No)**	**Proportion of studies with a control group**
Van Laethem et al 2013 (12) (Various job groups)	Strong quality review	Improvement of employees' influence, social support, and balance between demands and resources (3 out of 19 studies): 2 non-randomized controlled studies, 1 before and after study	Sleep quality	Only one of the three studies found a significant effect on sleep quality. However, in one of the intervention studies without effect, the intervention was not fully implemented.<br>(Meta-analysis: No)	High
Aust et al 2004 (13) (Various job groups)	Moderate quality review	Participatory interventions to improve the work environment (Health circles) (11 out of 11 studies): 3 non-randomized controlled studies, 8 before and after studies	Various psychosocial and physical work environment aspects, various health effects,	A tendency for positive effects was found, but studies of higher methodological quality are needed.<br>(Meta-analysis: No)	Medium

			job satisfaction, and sickness absence		
Bambra et al 2007 (14) (Various job groups)	Moderate quality review	Interventions that increase employees' control through task restructuring interventions (19 out of 19 studies): 10 controlled prospective cohort studies, 4 uncontrolled prospective cohort studies, 5 repeat cross-sectional studies	Various psychosocial work environment aspects, various health outcomes, and social support	Studies that increased control and decreased demands tended to result in improved health outcomes. Not all studies changed the psychosocial work environment and therefore no effect on health could be expected. Interventions that are conducted for economic reasons seem to have a tendency for negative health effects.<br>(Meta-analysis: No)	High
Egan et al 2007(15) (Various job groups)	Moderate quality review	Organizational-level interventions to increase employee control (18 out of 18 studies): 12 prospective nonrandomized controlled studies, 3 uncontrolled prospective, and 3 uncontrolled retrospective studies	Various psychosocial work environment aspects, various health outcomes, and social support	There was a tendency for positive health effects through improved control.<br>(Meta-analysis: No)	High

Conclusion

Together, the four reviews in this section covered 51 studies (three studies are included in more than one review). About half of these studies (27) had a control group, 0 RCTs.

⋆⋆ **Moderate quality of evidence:** Consistent results from multiple reviews with a medium proportion of studies with a control group or less consistent results from multiple reviews with a high proportion of studies with a control group.
Specifically: Three reviews with a high proportion of studies with a control group (Van Laethem et al 2013 (12), Bambra et al 2007 (14), Egan et al 2007(15)) and one review with a medium proportion of studies with a control group (Aust et al 2004 (13)) found that increased control can lead to positive health effects.

There is moderate quality of evidence that interventions that increase employee control can lead to positive health effects for employees. However, not all studies found positive results, which partly might be due to incomplete implementation. Interventions that were conducted for economic reasons seem to have a tendency for negative health effects.

3. Health care approach changes (3 reviews/32 studies)					
**Reference (Job groups)**	**Quality of review**	**Type of Intervention (number of studies relevant for this overview) and study design of relevant studies**	**Outcomes**	**Main results (Meta-analysis: Yes/No)**	**Proportion of studies with a control group**
Barbosa et al 2014 (29) (Health care staff)	Moderate quality review	Person-oriented dementia care (7 out of 7 studies): 4 RCT studies, 1 non-randomized controlled before and after study, 2 uncontrolled studies (before and after; repeated measure)	Stress, burnout, and job satisfaction	A tendency for positive effects was found (five studies reported benefits), but studies of higher methodological quality are needed. (Meta-analysis: No)	High
Elliot et al 2012 (30) (Health care staff)	Moderate quality review	To review the effects of dementia care training on health care workers capacity and organizational outcomes (6 out of 6 studies): 6 RCT studies	Health care workers' psychological wellbeing, work performance, and organizational factors (e.g. retention)	No clear conclusion could be drawn due to a mix of positive and null-effects, a lack of lasting effects and the poor scientific quality of the studies. (Meta-analysis: No)	High
Spector et al 2016 (31) (Health care staff)	Strong quality review	Staff training in dementia care (19 out of 19 studies): 11 RCT studies, 3 non-randomized quasi-experimental controlled studies, 5 quasi-experimental one-group time series studies.	Knowledge, burnout, job satisfaction, sense of competence, and self-efficacy	16 studies found improvement in at least one staff outcome. Knowledge increased most frequently (in six of seven interventions). (Meta-analysis: No)	High

Conclusion

Together, the three reviews in this section covered 32 studies (6 studies are included in more than one review). More than half of these studies (25) were studies with a control group, including 21 RCT studies (of which 4 are included in more than one review).

⋆⋆ **Moderate quality of evidence:** Consistent results from multiple reviews with a medium proportion of studies with a control group or less consistent results from multiple reviews with a high proportion of studies with a control group.
Specifically: Three reviews with a high proportion of studies with a control group (Barbosa et al 2014 (29)**,** Elliot et al 2012 (30)**,** Spector et al 2016 (31)**)** found some positive results for knowledge, burnout, stress, or job satisfaction, however also studies with null-effects and a lack of lasting effects were found.

There is moderate quality of evidence that interventions introducing health care approach changes can lead to improvements in employees' knowledge, burnout, stress, or job satisfaction.

4. Improvement of the psychosocial work environment (3 reviews/32 studies)					
**Reference (Job groups)**	**Quality of review**	**Type of Intervention (number of studies relevant for this overview) and study designs of relevant studies**	**Outcomes**	**Main results (Meta-analysis: Yes/No)**	**Proportion of studies with a control group**
Schalk et al 2010 (16) (Nurses)	Strong quality review	A variety of organizational-level interventions to improve the work environment of nurses (10 out of 11 studies): 2 randomized controlled studies, 8 non-randomized controlled studies	Various psychosocial work environment aspects (e.g. teamwork, leadership, autonomy, workload, workplace safety)	Inconclusive: Most of the interventions showed mixed effects (for most interventions some positive effects were found, however some outcomes where unchanged and in a few studies single negative outcomes were found). The most effective interventions were individualized care and clinical supervision, violence prevention, educational toolbox, and primary nursing). (Meta-analysis: No)	High
Daniels et al 2017 (17) (Various job groups)	Moderate quality review	Interventions that aimed to change the social environment at work, such as interventions to change perceived organizational support, organizational climate, social identity and organizational justice (8 out of 8 studies): 2 randomized controlled studies, 2 non-randomized controlled studies, 4 uncontrolled before and after studies	Indicators of wellbeing (mostly job satisfaction) and performance (but not performance alone)	Six out of six studies that introduced shared social activities in workgroups (e.g. dialogue groups, team building, internal mentoring programs) improved wellbeing. Two studies improving fairness perceptions showed inconsistent effects on wellbeing. (Meta-analysis: No)	High
Paguio et al 2019 (18) (Nurses)	Moderate quality review	Interventions directed at improving nurses' work environments (14 out of 14 studies): 4 quasi-experimental studies with control group, 10 quasi-experimental studies without control group	Nurse outcomes: work environment, job satisfaction, burnout, intention to leave, autonomy, and leadership. Patient outcomes: satisfaction, errors and falls. Hospital outcomes: workload, nurse turnover, and quality of care	Eight out of fourteen studies showed effects on at least one outcome (e.g. work environment, job satisfaction, autonomy, leadership, quality of care, patient satisfaction). These studies included elements of rapid improvement processes using participatory approach, running continuous quality improvement project, and developing action plans allowing tailoring of the content.	Medium

				(Meta-analysis: No)	

Conclusion

Together, the three reviews in this section covered 32 studies (0 studies are included in more than one review). More than half of these studies (18) had a control group, of which 4 studies were RCTs.

⋆⋆ **Moderate quality of evidence:** Consistent results from multiple reviews with a medium proportion of studies with a control group or less consistent results from multiple reviews with a high proportion of studies with a control group.
Specifically: Two reviews with a high proportion of studies with a control group (Schalk et al 2010 (16), Daniels et al 2017 (17)) and one review with a medium proportion of studies with a control group (Paguio et al 2019 (18)) found studies with positive results but not consistently.

There is moderate quality evidence that interventions that improve various aspects of the psychosocial work environment can lead to positive effects in the psychosocial work environment or employee wellbeing. Positive outcomes were found in studies that introduced workgroup activities focusing on better communication and support and in studies using a participative approach to enhance process aspects in the work environment and the core task.

5. Introduction programs for newly trained nurses (6 reviews/127 studies)

Reference (Job groups)	Quality of review	Type of Intervention (number of studies relevant for this overview) and study designs of relevant studies	Outcomes	Main results (Meta-analysis: Yes/No)	Proportion of studies with a control group
Chen et al 2014 (6) (Nurses)	Moderate quality review	Mentorship programs for recently registered nurses (5 out of 5 studies): 3 controlled before after studies, 2 uncontrolled before and after studies	Nursing competencies, job satisfaction, communication skills, development of interpersonal relationships, and turnover rates	Effective with regard to competence, job satisfaction, communication skills and development of interpersonal relationships and potentially for turn-over rate. (Meta-analysis: No)	High
Bakker et al 2020 (7) (Nurses)	Strong quality review	Interventions aimed at improving mental health of student or novice nurses and dropout-related outcomes (21 out of 21 studies): 2 RCT, 6 controlled trials, 1 controlled before and after study, 7 uncontrolled longitudinal studies, 4 uncontrolled post-test only studies, 1 cross-sectional study	Dropout related outcomes: Turnover, retention, intention to stay, sickness absence, and attrition	Five out of 21 studies showed an effect on dropout related outcomes. One study found negative effects, four no effect and 11 unclear effect (p-values not indicated or insufficient statistical methods used). (Meta-analysis: No)	Medium

Brook et al 2019 (8) (Nurses)	Moderate quality review	Interventions to promote retention and reduce turnover of early career nurses (53 out of 53 studies): 1 RCT study, 8 controlled before and after studies, 7 time series controlled studies, 8 controlled post-test only studies, 15 time series studies, 14 before and after not controlled studies	Attrition, retention and turnover	Do not report levels of significance but report that most of the studies decreased turnover and increased retention with averages between 9-24%. Few studies showed negative effects<br>(Meta-analysis: No)	Medium
Zhang et al 2016 (9) (Nurses)	Moderate quality review	Mentoring programs targeting newly graduated nurses (9 out of 9 studies): 1 RCT study, 1 controlled before and after study, 7 before and after studies	Turnover rate, cost-effectiveness, job satisfaction, nursing competency, and a category of "other outcomes"	Four studies reported significantly reduced turnover rate. Two studies showed reduced turnover costs. Four studies found mixed effects on job satisfaction. Three studies found improved competencies in newly educated nurses, two of them significant.<br>(Meta-analysis: No)	Low
Edwards et al 2015 (10) (Nurses)	Moderate quality review	Introduction programs for newly qualified nurses, such as nurse internship/residency programs, graduate nurse orientation programs, mentorship/preceptorship programs, and simulation-based graduate programs (28 out of 30 studies): 1 RCT study, 3 controlled before and after studies, 24 uncontrolled studies including longitudinal studies	Confidence, competency, knowledge, job satisfaction, stress, retention, and turnover	Positive effects for knowledge, competencies, confidence, stress, job satisfaction, retention, and turnover were found. However, due to the low methodological quality of most studies the findings of the review need to be confirmed by studies of higher quality.<br>(Meta-analysis: No)	Low
Missen et al 2014 (11) (Nurses)	Strong quality review	Programs to assist nursing graduates in transitioning from nursing student to advanced beginner in their first year of clinical practice including specific healthcare training, preceptorship or mentorship programs. (11 out of 11 studies): 1 controlled before and after study, 1 controlled posttest only study, 4 before and after studies, 2 prospective longitudinal studies, 3 descriptive comparative studies	Job satisfaction and/or confidence levels of nursing graduates	Seven studies found similar or slightly higher scores in satisfaction at 12 months. Eight studies found either increases in retention rate, ranging from 78–89% or decreases in turnover rate in the range of 4–12%.<br>(Meta-analysis: No)	Low
**Conclusion**<br>Together, the six reviews in this section covered 127 studies (11 studies are included in more than one review). About one third of these studies (44) had a control group, of which 5 studies were RCTs.<br><br>⋆ **Low quality of evidence**: Consistent results from multiple reviews with a low proportion of studies with a control group or less consistent results from multiple reviews with a medium proportion of studies with a control group.					

Specifically: Two reviews with a low proportion of studies with a control group (Zhang et al 2016 (9)**,** Edwards et al 2015 (10)) and one review with a high proportion of studies with a control group (**Chen et al 2014 (6)**) found positive effects for competencies.<br><br>**There is low quality of evidence that introducing newly educated nurses to their first job through mentoring programs or other forms of systematic and supportive introduction result in consistent results that it improves competencies. The intervention might also be effective with regard to decreasing turnover rate and improving job satisfaction, but results are less consistent.**

6. Prevention of workplace violence (4 reviews/68 studies)

**Reference (Job groups)**	**Quality of review**	**Type of Intervention (number of studies relevant for this overview) and study design of relevant studies**	**Outcomes**	**Main results (Meta-analysis: Yes/No)**	**Proportion of studies with a control group**
Price et al 2015 (25) (Mental health care staff)	Strong quality review	De-escalation techniques for management of violence and aggression (38 out of 38 studies): 3 case control studies, 12 controlled cohort studies, 23 uncontrolled cohort studies	Cognitive, affective, skills-based, clinical, and organizational outcomes	Positive effects were found for knowledge and confidence to manage aggression and de-escalation performance. The impact on assaults, injuries, containment, and organizational outcomes was less clear. However, more consistent effects were found at ward-level rather than individual-level (Meta-analysis: No)	Medium
Anderson et al 2010 (26) (Emergency department nurses)	Moderate quality review	Different types of interventions to prevent violence and threats from patients, including interventions that focused on the physical work environment, practices, and policies, or on employees' competencies (10 out of 10 studies): 3 controlled before and after studies, 3 before and after studies, 1 post-test only study, 3 uncontrolled cross-sectional studies	Feeling safe, physical safety equipment, reporting, competencies, violence from patients	Although some positive effects were found in almost all studies, the authors conclude that the quality and the approach of the different studies are too varied to reach a firm conclusion about which strategies work best. (Meta-analysis: No)	Medium
Tölli et al 2017 (27) (Nursing staff)	Moderate quality review	Training intervention to enhance competence of staff when managing challenging behavior (17 out of 17 studies, with 16 samples): 4 RCT studies, 2 controlled before and after studies, 11 interrupted time series design studies	Violence incidents, staff confidence, staff attitudes, and knowledge	Interventions were more likely to decrease violent incident rates and increase staff confidence than change staff attitudes or increased knowledge. (Meta-analysis: No)	Medium

Kynoch et al 2011 (28) (Acute hospital staff)	Moderate quality review	Education and training in managing aggressive behaviors (3 out of 10 studies): 1 controlled prospective study, 2 uncontrolled before and after studies	Knowledge, confidence, competencies, attitudes, and violence incidents	All three studies found significant improvements in knowledge, confidence, and competencies.<br>(Meta-analysis: No)	Medium

Conclusion

Together, the four reviews in this section covered 68 studies (5 studies are included in more than one review). Less than half of these studies (27) were studies with a control group, including 4 RCT studies.

Three reviews with a medium proportion of studies with a control group found positive effects of workplace violence prevention interventions on employees' confidence, knowledge, and competencies.

With regard to effects for violence
⋆ **Low quality of evidence:** Consistent results from multiple reviews with a low proportion of studies with a control group or less consistent results from multiple reviews with a medium proportion of studies with a control group

Specifically: Three reviews (Kynoch et al, 2011 (28); Price et al, 2015 (25); Tölli et al, 2017 (27)) found that staff training can improve confidence, while Kynoch et al (2011) (28) and Price et al (2015) (25) found that it can also increase knowledge. However, with regard to decreasing violence we found low quality evidence. Two reviews with a medium proportion of studies with a control group found less consistent positive results: one review found less clear results for violence reduction compared to other outcomes (Price et al, 2015 (25)) while another review found more clear results for violence reduction compared to other outcomes (Tölli et al, 2017 (27)).

There is low quality of evidence that workplace violence prevention interventions can decrease violence.

7. Leadership training or development (6 reviews/310 studies)

**Reference (Job groups)**	**Quality of review**	**Type of Intervention (number of studies relevant for this overview) and design of relevant studies**	**Outcomes**	**Main results (Meta-analysis: Yes/No)**	**Proportion of studies with a control group**
Collins et al 2004 (19) (Various job groups)	Moderate quality review	Various leadership development interventions (103 studies were found, but only 83 consisted of a formal leadership training and only these studies were used in the meta-analysis) (83 out of 103 studies): 26 pretest-posttest with control studies, 36 posttest only with control studies, 25	Knowledge, competencies, and organizational aspects (such as employees' job satisfaction)	The meta-analysis showed positive effects for knowledge and competencies, but only moderate effects for organizational aspects (which were measured in fewer studies).	High

		single group pretest-posttest studies. They do not report why numbers for study design do not add up.		The effects of the included studies varied a lot.<br>(Meta-analysis: Yes)	
Grover et al 2016 (20) (Various job groups)	Moderate quality review	Executive, leadership and business coaching (52 out of 52 studies, based on 46 samples): 3 RCT studies, 21 controlled studies (4 of which are retrospective), 22 uncontrolled studies (12 of which are retrospective)	Self-efficacy, mental health and wellbeing, satisfaction and performance, other outcomes, and transformational leadership, subordinates satisfaction, work engagement, psychological strain, and turnover intentions	Coaching can affect the coachees and their employees on several outcomes, but there was a considerable variation in outcomes measured and effects<br>(Meta-analysis: No)	High
Gayed et al 2018 (21) (Various job groups)	Strong quality review	Workplace interventions for managers with an emphasis on the mental health of employees reporting directly to them (9 out of 9 articles, based on 10 studies): 9 RCT studies, 1 controlled before and after study	Managers' mental health knowledge, non-stigmatizing attitudes towards mental health, behavior supporting employees experiencing mental health problems, and psychological symptoms in employees	Interventions showed effects on managers' knowledge, attitudes, and behavior, but not on psychological symptoms in employees<br>(Meta-analysis: Yes)	High
Stuber et al 2021 (22) (Health care sector)	Strong quality review	Leadership interventions aimed to maintain/foster employees' mental health (7 out of 7 studies): 1 RCT study, 3 controlled clinical trials, 1 study with cohort analytic design, 2 uncontrolled studies	Mental health of employees, mental health of leaders	Two of the four studies that measured mental health of employees found improvements, and two of the three studies that measured leader mental health found improvements.<br>(Meta-analysis: No)	High
Kuehnl et al 2019 (23) (Various job groups)	Strong quality review	Off-the-job supervisor training (face-to-face lectures, simulations, role-playing), on-the-job supervisor training (personal coaching, feedback sessions) (21 out of 21 studies): 15 RCT studies, 6 controlled before and after studies	Validated measures of psycho-mental stress, absenteeism, wellbeing or work engagement. All outcomes assessed in employees	The review found inconsistent evidence that supervisor training may or may not improve employees' wellbeing when compared to no intervention. Studies with better designs are needed to further investigate these aspects.<br>(Meta-analysis: Yes)	High
Avolio et al 2009 (24)	Moderate quality review	Leadership training and development interventions (138 out of 138 studies): 61	Aggregation of affective, behavioral, and cognitive outcomes in leaders	The leadership interventions had a 66% chance for a positive outcome compared to 34% for	Medium

(Various job groups)	controlled studies and 56 uncontrolled studies. For the last 21 studies design was not reported.	the control group. Despite this positive effect of leadership interventions, the study effects were very heterogeneous. (Meta-analysis: Yes)

Conclusion

Together, the six reviews in this section covered 310 studies (6 studies are included in more than one review. However, overlap could not be calculated for Avolio since they report no reference list of included studies). Almost 60% of these studies (182) were studies with a control group, of which 13 were RCTs.

Inconclusive evidence due to contradictory results: Contradictory results from multiple reviews with a medium or high proportion of studies with a control group
Specifically: One review with a high proportion of studies with a control group did not find effects on psychological symptoms for employees (Gayed et al, 2018 (21)), while four reviews with a high proportion of studies with a control group found mixed results for employees' mental health (Stuber et al, 2020 (22)) or wellbeing (Kuehnl et al, 2019 (23)), or moderate effects for organizational aspects (such as employees' job satisfaction) (Collins et al, 2004 (19)) or varying effects for employees' work environment or health outcomes (Grover et al, 2016 (20)).

There is inconclusive evidence due to contradictory results for interventions of leadership trainings and development can lead to positive health and work environment effects for employees.

e-Table 5.2: Synthesis of the 22 reviews that examined interventions with focus on employees' health, wellbeing, or labor market retention

**1. Burnout (8 reviews/125 studies)**					
**Reference (Job groups)**	**Quality of review**	**Type of Intervention (number of studies relevant for this overview) and design of relevant studies**	**Outcomes**	**Main results (Meta-analysis: Yes/No)**	**Proportion of studies with a control group** High: More than 50% studies with a control group Medium: 50% - 25% Low: Less than 25%
Panagioti et al 2016 (32) (Physicians)	Moderate quality review	Individual and organizational directed interventions to reduce physician burnout (7 out of 19 studies): 7 RCT studies	Burnout	All interventions showed small significant reductions in burnout, and subgroup analysis suggested significantly improved effects for organization-directed interventions compared with physician-directed interventions. Organizational directed interventions that combined several elements such as structural changes, fostering communication between members of the health care team, and cultivating a sense of teamwork and job control tended to be the most effective in reducing burnout, but only few interventions were that comprehensive. The majority of organization-directed interventions introduced simple reductions in the workload or schedule changes.<br><br>(Meta-analysis: yes)	High
Awa et al 2010 (33)	Moderate quality review	Various interventions, including organizational and combination of organizational and individual changes	Burnout	Inconsistent results from exclusively organizational focused interventions (2 studies), but consistent positive results from	High

(Various job groups)		aimed at preventing burnout (8 out of 25 studies): 4 RCT studies, 2 controlled non randomized studies, and 2 non-controlled observational studies		interventions combining individual with organizational approaches (6 studies). The content of these interventions differed between studies but included for example professional supervision, work schedule, reorganization, lectures, reorganization, communication, feedback, participatory action research (PAR) communication, social support and improved coping skills.<br>(Meta-analysis: No)	
Dreison et al 2016 (34) (Mental health providers)	Strong quality review	Individual and organizational directed interventions to reduce burnout (21 out of 27 samples from 29 studies): 10 RCT studies and 11 uncontrolled studies	Burnout	All interventions showed small significant reductions in burnout. Subgroup analysis suggested significantly improved effects on the personal exhaustion dimension for person-directed interventions compared with organization-directed interventions, but not on other dimensions. Job training and education were the most effective organizational intervention subtype.<br>(Meta-analysis: yes)	Medium
Pijpker et al 2019 (35) (Various job groups)	Moderate quality review	Combined individual and organizational-directed interventions (10 out of 10 studies): 1 RCT study, 4 controlled not randomized studies, and 5 uncontrolled studies	Burnout, return to work	All interventions included were effective in facilitating rehabilitation defined as reduced burnout complaints and full return to work. Moderator analysis showed that enhanced job-control, social support and elimination of stressors explain the effectiveness of interventions.<br>(Meta-analysis: no)	Medium
DeChant et al 2019 (36) (Physicians)	Moderate quality review	Organizational-directed interventions related to teamwork, time, workflow changes, policy changes, and technology (50 out of 50 studies): 10 RCT studies, 24 before-after studies with no control, 5 prospective studies, 7 cross-sectional studies, and 4 other designs (including 3 controlled studies)	Burnout, job satisfaction, and stress	70% of the included studies showed improvements in the three measures of physician burnout, job satisfaction, and /or stress. Interventions differed a lot. Evidence from high quality studies suggests that streamlining workflows, providing leadership-driven professional support opportunities, and reducing the administrative burden of EHRs	Medium

				(electronic health records) through team based care by the use of scribes and medical assistants generally improve physician burnout. (Meta-analysis: No)	
Xu et al 2020 (37) (Emergency department staff)	Strong quality review	Organizational-level interventions to reduce stress and/or burnout in emergency department staff (4 out of 14 studies): 1 controlled study, 3 studies without a control group	Three of the four studies measured burnout, the fourth study measured work related stress	One study focused on reducing stress and found a significant reduction in stress. Of the three studies that measured burnout, one found no differences, while two studies found that burnout levels worsened after the intervention. Worsened results might be due to limited support from the organization to conduct actual changes in the working environment. (Meta-analysis: No)	Medium
West et al 2016 (38) (Physicians)	Strong quality review	Individual and organizational-directed interventions to reduce physician burnout (20 out of 52 studies): 3 RCT studies and 17 cohort studies	Burnout	Both individual and organizational directed interventions showed significant reductions in different burnout domains but not in the overall burnout. Among the organizational directed interventions especially small group discussions and duty hour limitation policies also appear to be effective for reducing burnout. (Meta-analysis: yes)	Low
Williams et al 2018 (39) (Health care organizations)	Moderate quality review	Interventions to improve employee health and wellbeing within health care organizations. Only the five studies in the category “changing ways of working” were considered here. However, the authors concluded only on the three studies with a “fair” methodological quality (5 (3) out of 44 studies). Study designs of relevant studies: 5 pre-post intervention studies with no control group	Any outcomes that fall into the domain of employee health and wellbeing. The three organizational-level interventions measured emotional exhaustion, work-related exhaustion and need for recovery	Interventions that involve employees in discussions about finding solutions to workplace problems and challenges were effective for subscales of burnout or related measures. (Meta-analysis: No)	Low
**Conclusion**<br>Together, the eight reviews in this section covered 125 organizational or combined organizational and individual studies (19 studies are included in more than one review).					

Five of the 19 studies were used in 3 reviews, while 14 studies were used in 2 reviews. The most overlap between two reviews (9 studies) were between the review by DeChant et al 2019 (36) and West et al 2016 (38) which both are reviews about burnout interventions among physicians. About a third of the 125 studies (38) were studies with a control group, of which 35 were RCTs.

⋆⋆⋆ **Strong quality of evidence:** Consistent results from multiple reviews with high or moderate quality and with a high proportion of studies with a control group. Specifically: Two reviews (Panagioti et al 2016 (32)**,** Awa et al 2010 (33)) with a high proportion of studies with a control group found that interventions that either are exclusively organizational or that use a combination of individual and organizational components can reduce burnout. This finding is supported by several other reviews with a medium or low proportion of studies with a control group.

There is strong quality of evidence that organizational-level interventions either by themselves or in combination with individual intervention components can reduce burnout. There was a large variety among the organizational-level interventions that were identified in the eight reviews, but several reviews pointed out which kind of changes were important for these changes. These were interventions that focus on work schedules, workload reductions and improved work organization, enhanced job-control and participation, social support, communication and feedback, supervision and leadership support.

2. Various health and wellbeing outcomes (6 reviews/83 studies)

**Reference (Job groups)**	**Quality of review**	**Type of Intervention (number of studies relevant for this overview) and design of relevant studies**	**Outcomes**	**Main results (Meta-analysis: Yes/No)**	**Proportion of studies with a control group studies with a control group**
Montano et al 2014 (40) (Various job groups)	Moderate quality review	Various interventions aiming to improve employees' health, including organizational, psychosocial, and physical changes to improve employees' health, 12 studies introduced changes in the material and/or organizational conditions, 16 studies concentrated on organizational conditions, eight studies emphasized on the time conditions of work and 3 studies comprised all three types of working conditions (39 out of 39 studies): 6 RCT studies, 21 prospective	Various health outcomes (including measures for physical and mental health) Examples are musculoskeletal disorders of the upper body, sleep quality, general health, somatic symptoms, stress, burnout, depressive symptoms, but also absenteeism	Overall inconsistent results, although about half of the studies showed significant effects on employees' health. There was a tendency for a higher chance of finding positive health effects in more comprehensive interventions (especially those that simultaneously tackled material, organizational, and work-time related conditions). (Meta-analysis: No)	High

		studies with control group, 9 one-group prospective studies, and 1 cross sectional study, 2 quasi-experimental prospective and retrospective studies			
Corbiere et al 2009 (41) (Various job groups)	Moderate quality review	Various interventions aiming at improving wellbeing and preventing mental health problems, including communication, organizational, and combination of organizational and individual changes. Many studies used skill training. (24 out of 24 studies): 11 RCT studies, 10 studies with a control group, and 3 uncontrolled studies	Various mental health related and work related outcomes. Examples are absenteeism, aspects of the psychosocial work environment, depressive symptoms, burnout, wellbeing, stress, work-life balance	Two thirds of the studies had positive effects on mental health outcomes and 60% of the 17 studies that measured it had positive effects on work related outcomes (perceived job characteristics and work environment aspects). Interventions combining individual, group, and organizational-level interventions showed several significant improvements in work and mental health-related outcomes, but this finding needs to be confirmed by studies of better quality. (Meta-analysis: No)	High
Gilbody et al 2006 (42) (Employees in psychiatry)	Strong quality review	Various interventions aiming to improve morale and wellbeing including improvement in communication, increasing social support, organizational changes (8 out of 8 studies): 3 RCT studies, 3 controlled clinical trials, and 2 controlled before and after studies	Psychological wellbeing, job satisfaction, staff turnover, sickness absence, costs, and burnout	Almost all studies found significant improvements in at least one of the outcomes. The one study without effect reported implementation problems. However, many studies had a small sample size and other methodological shortcomings. (Meta-analysis: No)	High

Romppanen et al 2016 (43) (Nurses)	Strong quality review	All types of interventions aiming at improving wellbeing at work were covered in the review, but here only combined person- and organizational-level interventions and solely organizational-level interventions were considered (7 out of 10 studies): 2 RCT studies, 4 controlled before and after studies, and 1 study with interrupted time series	Wellbeing at work defined as summative concept that includes a large range of personal level (e.g. health), work level (e.g. communication), and organizational-level (e.g. absence) outcomes	Six of the seven studies led to some positive effects; however, in one combined person- and organizational-level study the effects were restricted to the most long-lasting part of the intervention. For one type of organizational-level intervention (clinical supervision) one study found effects, the other did not.<br>(Meta-analysis: No)	High
Lee et al 2014 (44) (Male-dominated workplaces)	Moderate quality review	Various interventions aiming at improving mental health, including social support, supervisor training, employee involvement (3 out of 5 studies): 2 RCTs, 1 case study	Mental health, sickness absence, and job performance	All three studies found positive effects in at least one of the outcomes, however, in one study effects were only found in the high-risk subgroup.<br>(Meta-analysis: No)	High
van Wyk et al 2010 (45) (Health care sector)	Strong quality review	Interventions that aimed to help health professionals to manage stress at work, prevent burnout or improve job satisfaction. Only 2 organizational-level studies were found. One on training employees in handling organizational changes and one on improving problem solving skills of supervisors (2 out of 10 studies): 2 RCT studies	Stress indicators, job satisfaction, and sickness absence	The one employee training study that studied that found positive effects on job satisfaction, but not on stress. The study on supervisor training measured sickness absence in employees but found no effect.<br>(Meta-analysis: No)	High

Conclusion

Together, the six reviews in this section covered 83 studies (9 studies are included in more than one review).
Each of these 9 studies were used in two out of the six reviews, none of them in more than two reviews. The most overlap of studies was between the reviews from Montano et al 2014 and Corbiere et al 2009 (6 studies were used in both of these reviews). The majority of the 83 studies (66) were studies with a control group, of which 26 were RCTs.

⋆⋆ **Moderate quality of evidence:** consistent results from multiple reviews with a medium proportion of studies with a control group or less consistent results from multiple reviews with a high proportion of studies with a control group.
Specifically: Six reviews with a high proportion of studies with a control group (i.e. all of the reviews in this group) found that at least half of the intervention studies led to some positive effects. In a few cases, the positive effects were only found for those that were at high risk (Lee et al 2014 (44)) or those that received a high dose of the intervention (Romppanen et al 2016 (43)). Two of the six reviews found a tendency for better effects from combined individual and organizational-level interventions (Corbiere et al 2009 (41)) or from more comprehensive interventions tackling many organizational aspects at once (Montano et al 2014(40)).

There is moderate quality of evidence that organizational-level interventions that aim to improve employees' various health and wellbeing outcomes can lead to positive effects. The interventions covered in the six reviews focusing on various health and wellbeing outcomes used a variety of approaches. The effective interventions included working time related changes, improvement in communication, increasing social support, organizational changes, clinical supervision, social support and employee involvement.

**3. Stress (6 reviews/47 studies)**					
**Reference (Job groups)**	**Quality of review**	**Type of Intervention (number of studies relevant for this overview)**	**Outcomes**	**Main results (Meta-analysis: Yes/No)**	**Proportion of studies with a control group**
Richardson et al 2008 (48) (Various job groups)	Moderate quality review	Various interventions with the aim to prevent stress reactions (e.g. participation in decision making, Social support groups) (5 out of 36 studies): 5 studies with random assignment to treatment and control conditions	Stress indicators, including psychological (most common), physiological, and organizational outcomes	The meta-analysis showed no overall effect for the five organizational-level interventions. (Meta-analysis: Yes)	High
van der Klink et al 2001 (49) (Various job groups)	Moderate quality review	Various interventions with the aim to reduce occupational stress (e.g. participation in decision making, employee committees, increase individual and group psychosocial coping resources) (5 out of 48 studies): 5 high quality experimental or quasi-experimental studies with control groups	The outcome variables covered five categories: quality of work (e.g. job demands, job control, social support), psychological resources and responses (e.g. coping skills), physiology (e.g. cholesterol), complaints (e.g. stress, burnout), and absenteeism. For the meta-analysis an overall effect based on these outcomes was calculated	The meta-analysis showed no overall effect for the five organizational-level interventions. (Meta-analysis: Yes)	High
Giga et al 2003 (50) (Various job groups)	Moderate quality review	Various interventions with a focus on organizational changes in distribution of work tasks, communication, and increased influence at work (9 out of 16 studies): 4 RCT studies, 4 studies with a control group but no randomization, and 1 cross-sectional study	Stress indicators and organizational outcomes (e.g. productivity)	Some positive effects were found in all types of interventions. Individual-level interventions were less likely to result in long-term benefits. (Meta-analysis: No)	High

Ruotsalainen et al 2015 (51) (Health care sector)	Strong quality review	Organizational-directed interventions aimed at changing working conditions including improving support or mentoring, changing content of care, improving communication skills, and improving work schedules (21 out of 56 studies): 16 RCT studies, and 5 controlled before and after studies	Stress indicators and burnout	Low quality evidence for the two studies about improving working time schedules. Not effective for other types of organizational-level interventions, but this is only based on one or two studies in each category (Meta-analysis: Yes)	High
Naghieh et al 2015 (52) (School teachers)	Strong quality review	Various interventions, all combining organizational with individual changes (changing task or organizational characteristics, multi-component intervention) (4 out of 4 studies). Study designs of relevant studies: 3 cluster RCT studies and 1 study with stepped-wedge design	Wellbeing, stress indicators, and workplace retention	Some weak evidence that organizational changes were effective. However, all studies combined organizational changes with individual changes. (Meta-analysis: No)	High
Mimura et al 2003 (53) (Health care sector)	Strong quality review	Interventions that supported nurses in handling stressors and that changed the work environment to reduce stressors (e.g. providing structured training and regular support, primary nursing in hospital wards, individualized nursing care) (3 out of 10 studies): 3 prospective cohort studies	Stress indicators and burnout	The three studies all showed some tendencies or potentials for positive effects, but due to too few studies it was not possible to draw a firm conclusion. (Meta-analysis: No)	Low

Conclusion

Together the 6 reviews in this section covered 47 studies (6 studies are included in more than one review). Each of these 6 studies were used in two out of the six reviews, none of them in more than two reviews. The most overlap of studies was between the reviews from Mimura et al 2002 and Ruotsalainen et al 2015 (2 studies were used in both of these reviews). The majority of the studies (43 of 47) were studies with a control group, of which at least 28 were RCT studies (one review (van der Klink) did not provide detailed information about this aspect).

Inconclusive evidence due to contradictory results: Contradictory results from multiple reviews with a medium or high proportion of studies with a control group. Specifically: Two (Richardson et al, 2008 (48); van der Klink et al, 2001 (49)) out of five reviews with a high proportion of studies with a control group found that organizational-level interventions to reduce stress had no effects. One review (Ruotsalainen et al 2015 (51)) found effects for only one specific type of intervention (scheduling), one review found that positive effects from organizational interventions lasted longer than from individual interventions (Giga et al, 2003 (50)), and one review found weak evidence for combined interventions (Naghieh et al 2015 (52)).

There is contradictory evidence about the ability of organizational-level interventions to reduce stress. The six reviews that focused on interventions to reduce stress covered a variety of interventions including participation in decision making, increased influence at work, social support groups, employee committees, improving support or mentoring, improving communication skills, organizational changes in distribution of work tasks, reduce stressors, changing content of care, improving work schedules, combination of organizational with individual changes.

4. Retention (2 reviews/6 studies)

**Reference (Job groups)**	**Quality of review**	**Type of Intervention (number of studies relevant for this overview)**	**Outcomes**	**Main results (Meta-analysis: Yes/No)**	**Proportion of studies with a control group**
Cloostermans et al 2015 (46) (Whole review: various job groups, for the only included study: Employees from an electronic equipment company)	Strong quality review	Assessment of employees' health, work modification, talks between employees and supervisors, and referral to physicians if needed (1 out of 4 studies): 1 RCT study	Early retirement, workability	Significant positive effects on retention. After 2 years, more workers in the control groups took early retirement. There were also positive effects for work ability after 6 months, however no longer after 2 years. Despite the positive effects, more studies are needed for a clear conclusion. (Meta-analysis: No)	High
Lartey et al 2014 (47) (Nurses with high seniority)	Moderate quality review	Various interventions to retain nurses at work, including new care approaches, changes in leadership style, and in organizational strategies (5 out of 12 studies, seven studies were correlational). Most of the 12 studies were non-experimental. Four of the 12 studies used a pre-post design to collect data and two had comparison groups. The review does not give more specific information on the study design of each study	Workplace retention	Although most studies found positive effects, more studies are needed for a clear conclusion. Retention was highest when multiple interventions were combined. (Meta-analysis: No)	Low

Conclusion

Together these two reviews identified 6 studies (0 studies are included in more than one review).

Inconclusive evidence due to lack of studies: High or moderate quality systematic reviews/meta-analyses that cannot draw conclusions due to limited number of studies. Specifically: Although all six identified studies show some positive effects on retention, both reviews do not reach a conclusion due to too few studies. Both reviews pointed out that interventions that initiate multiple strategies at the same time may be better suited to sustaining older employees.

There is inconclusive evidence due to lack of studies about the effect of organizational-level interventions on employee retention.

e-Appendix 6: Overlap between primary studies in the three review groups about burnout, various health and wellbeing outcomes and stress.

Reviews that focus on burnout

The eight reviews that focused on the outcome burnout included a total of 125 primary studies. Of these, 19 studies (15.2%) were included in more than one review. Five of the 19 studies were used in three reviews, while 14 studies were used in two reviews. The most overlap between two reviews (9 studies) were between the review by DeChant et al 2019 (36) and West et al 2016 (38) which both were reviews about burnout interventions among physicians.

e-Table 6.1: Overlap of primary studies in reviews on burnout

**Reviews that focus on burnout**	Panagioti et al 2016 (32) (Physicians)	Awa et al 2010 (33) (Various job groups)	Dreison et al 2016 (34) (Mental health providers)	Pijpker et al 2019 (35) (Various job groups)	DeChant et al 2019 (36) (Physicians)	Xu et al 2020 (37) (Emergency department staff)	West et al 2016 (38) (Physicians)	Williams et al 2018 (39) (Health care organizations)	**How often was that study used?**
**Primary studies that are included in more than one review**						No overlap			
1. Ali et al 2011	X				X				2
2. Blonk et al 2006		X		X					2
3. Dunn et al 2007					X		X	X	3
4. Garland et al, 2012	X				X				2
5. Giannini et al 2013					X		X		2
6. Hill et al 2010			X	X					2
7. Innstrand et al 2004		X		X					2
8. Landrigan et al 2008					X		X		2
9. Le Blanc et al 2007		X		X					2
10. Linzer et al 2015	X				X		X		3
11. Lucas et al 2012	X				X		X		3
12. Melchior et al 1996		X	X						2
13. Parshuram et al 2015	X				X		X		3
14. Quenot et al 2012					X		X		2
15. Ripp et al 2015					X		X		2
16. Schuh et al 2011					X		X		2
17. Shea et al 2014	X				X				2
18. Sluiter et al 2005		X					X	X	3
19. West et al 2014	X				X				2

Reviews with that focus on various health and wellbeing outcomes

The six reviews that focused on various health and wellbeing outcomes included a total of 83 primary studies. Of these, nine studies (10.8%) were included in more than one review. Each of these nine studies were used in two of the six reviews, none of them in more than two reviews. The most overlap of studies was between the reviews from Montano et al 2014 (40) and Corbiere et al 2009 (41) (six studies were used in both of these reviews).

e-Table 6.2: Overlap of primary studies in reviews on various health and wellbeing outcomes

**Reviews with a focus on various health and wellbeing outcomes**	Montano et al 2014 (40) (Various job groups)	Corbiere et al 2009 (41) (Various job groups).	Gilbody et al 2006 (42) (Employees in psychiatry)	Romppanen et al 2016 (43) (Nurses)	Lee et al 2014 (44) (Male-dominated workplaces)	van Wyk et al 2010 (45) (Health care sector)	**How often was that study used?**
**Primary Studies that are used in more than one review**				No overlap		No overlap	
1. Bond et al 2001	**X**	**X**					2
2. Bourbonnais et al 2006	**X**	**X**					2
3. Ewers et al 2002		**X**	**X**				2
4. Logan et al 2005	**X**	**X**					2
5. Mattila et al 2006	**X**	**X**					2
6. Melchior 1996	**X**		**X**				2
7. Pryce et al 2006	**X**	**X**					2
8. Schrjinemaekers et al 2003	**X**	**X**					2
9. Tsutsumi et al 2009	**X**				**X**		2

Reviews that focus on stress

The six reviews that focused on the outcome stress included a total of 47 studies. Of these, six studies (12.8%) were included in more than one review. Each of these six studies were used in two out of the six reviews, none of them in more than two reviews. The most overlap of studies was between the reviews from Mimura et al 2002 (53) and Ruotsalainen et al 2015 (51) (two studies were used in both of these reviews).

e-Table 6.3: Overlap of primary studies in reviews on stress

**Reviews that focus on stress**	Richardson et al 2008 (48) (Various job groups	van der Klink et al 2001 (49) (Various job groups)	Giga et al 2003 (50) (Various job groups)	Ruotsalainen et al 2015 (51) (Health care sector)	Naghieh et al 2015 (52) (School teachers)	Mimura et al 2003 (53) (Health care sector)	**How often was that study used?**
**Primary studies that were used in more than one review**					No overlap		
1. Bond et al 2000	**X**		**X**				2
2. Carson et al 1999	**X**			**X**			2
3. Heaney et al 1995		**X**		**X**			2
4. Jackson et al 1983	**X**	**X**					2
5. Melchior et al 1996				**X**		**X**	2
6. Proctor et al 1998				**X**		**X**	2

e-Appendix 7: Levels of quality of evidence

Levels of quality of evidence

⋆ ⋆ ⋆ **Strong quality of evidence**
High or moderate quality systematic reviews/meta-analyses demonstrating consistent results from multiple reviews with a high proportion of studies (>50%) with a control group.

⋆⋆ **Moderate quality of evidence**
High or moderate quality systematic reviews/meta-analyses demonstrating consistent results from multiple reviews with a medium proportion of studies (25% to 50%) with a control group or less consistent results from multiple reviews with a high proportion of studies (>50%) with a control group**.**

⋆ **Low quality of evidence**
High or moderate quality systematic reviews/meta-analyses demonstrating consistent results from multiple reviews with a low proportion of studies (<25%) with a control group or less consistent results from multiple reviews with a medium proportion of studies (25% to 50%) with a control group.

Inconclusive evidence due to lack of studies
High or moderate quality systematic reviews/meta-analyses that cannot draw conclusions due to limited amount of studies.

Inconclusive evidence due to contradictory results
High or moderate quality systematic reviews/meta-analyses demonstrating contradictory results from multiple reviews with a medium (25% to 50%) or high proportion (>50%) of studies with a control group.

Consistent results: almost all studies that measure that effect show an effect in the same direction (or show consistently the absence of an effect)

Less consistent results: only some of the studies that measure that effect show an effect in the same direction.

e-Table 7.1: Illustration of the levels of quality of evidence

	Quality of the review	Consistency of results	Proportion of studies with a control group	Reason for inconclusive evidence	Evidence level for the different groups of reviews
	High or moderate quality systematic reviews/meta-analyses	Consistent results (almost all studies that measure that effect show an effect in the same direction (or show consistently the absence of an effect) / less consistent results (only some of the studies that measure that effect show an effect in the same direction)	High: More than 50% studies with a control group<br>Medium: 50% - 25%<br>Low: Less than 25%	Lack of studies<br>or<br>contradictory results	
⋆ ⋆ ⋆ **Strong quality of evidence**	Multiple reviews	Consistent results	High proportion of studies with a control group		• Changes in working time arrangements (group 1)<br>• Burnout (group 2)
⋆⋆ **Moderate quality of evidence**	Multiple reviews	Consistent results	Medium proportion of studies with a control group		• Influence on work tasks or work organization (group1)
⋆⋆ **Moderate quality of evidence**	Multiple reviews	Less consistent results	High proportion of studies with a control group		• Health care approach changes (group 1)<br>• Improvement of the psychosocial work environment (group 1)<br>• Various health and wellbeing outcomes (group 2)
⋆ **Low quality of evidence**	Multiple reviews	Consistent results	Low proportion of studies with a control group		• Introduction programs for newly trained nurse (group 1)
⋆ **Low quality of evidence**	Multiple reviews	Less consistent results	Medium proportion of studies with a control group		• Prevention of workplace violence (group 1)
**Inconclusive evidence due to lack of studies**	Multiple reviews	Too few studies to reach a conclusion	Too few studies to assess	Reviews cannot draw conclusions due to lack of studies	• Retention (group 2)
**Inconclusive evidence due to contradictory results**	Multiple reviews	Reviews that consistently find no effects and reviews that find consistent or less consistent positive results	High or medium proportion of studies with a control group	Contradictory results	• Leadership training or development (group 1)<br>• Stress (group 2)

e-Appendix 8: List of the 61 reviews excluded from the overview of reviews with reason

Reports excluded with reason n = 61

Not a systematic review of single studies n = 11 (e.g. no quality assessment or scooping review)

1. Ahola et al 2017 (54)
2. Hayman-White et al 2007 (55)
3. Levett-Jones et al 2005 (56)
4. Morse et al 2012 (57)
5. Park et al 2010 (58)
6. Paris et al 2010 (59)
7. Thomas 2015 (60)
8. Tsutsumi 2011 (61)
9. Verbeek et al 2019 (62)
10. Weissbrodt et al 2017 (63)
11. Zhang et al 2020 (64)

Not intervention studies n = 4

1. Alilyyani et al 2018 (65)
2. Cummings et al 2018 (66)
3. Hall et al 2016 (67)
4. McVicar et al 2013 (68)

Not organizational-level interventions n = 8

1. Clough et al 2017 (69)
2. Edwards et al 2003 (70)
3. Maricutoi et al 2016 (71)
4. Michie et al 2003 (72)
5. Nowrouzi et al 2015 (73)
6. Oprea et al 2019 (74)
7. Ouellette et al 2020 (75)
8. Regehr et al 2014 (76)

Not about psychosocial work environment n = 7

1. Anger et al 2015 (77)
2. Frich et al 2015 (78)
3. Jones et al 2016 (79)
4. Knight et al 2017 (80)
5. Knight et al 2019 (81)
6. Rapaport et al 2017 (82)
7. Shultz et al 2015 (83)

Not a separate conclusion about quantitative organizational-level intervention studies n = 30

1. Armstrong 2018 (84)
2. Bartlett et al 2019 (85)
3. Brand et al 2017 (86)
4. Buljac-Samardzic et al 2010 (87)
5. Chesak et al 2019 (88)
6. Cooklin et al 2017 (89)
7. Czabala et al 2011 (90)
8. De Oliveira et al 2019 (91)
9. Duhoux et al 2017 (92)
10. Elder et al 2020 (93)
11. Francke et al 2012 (94)
12. Kuoppala et al 2008 (95)
13. LaMontagne et al 2007 (96)
14. Martin et al 2009 (97)
15. McCray et al 2008 (98)
16. Meyers et al 2013 (99)
17. Moran et al 2014 (100)
18. Murray et al 2016 (101)

19. Niskala et al 2020 (102)
20. O’Donovan et al 2020 (103)
21. Stewart et al 2014 (104)
22. Steinert et al 2012 (105)
23. Suter et al 2012 (106)
24. Tan et al 2014 (107)
25. Taylor et al 2018 (108)
26. van Mol et al 2015 (109)
27. Vîrgă et al 2021 (110)
28. Viselita et al 2019 (111)
29. Westgaard et al 2011 (112)
30. Wiederhold et al 2018 (113)

Redundant n = 1

1. De Simone et al 2021 (114)

e-Appendix 9: Quality assessment of the 76 reviews with strong, moderate or weak quality

Quality assessment question / Author (year)	Q1: Are the population, intervention and outcome clearly described in the research question or inclusion criteria?	Q2: Were appropriate inclusion criteria used to select primary studies?	Q3: Did the authors describe a search strategy that was comprehensive?	Q4: Did the search strategy cover an adequate number of years?	Q5: Did the authors describe the level of evidence in the primary studies included in the review?	Q6: Did the review assess the methodological quality of the primary studies?	Q7: Are the quality of the primary studies assessed by a minimum of two authors and the method of conflict resolution described?	Q8: Was it appropriate to combine the findings of results across studies?	Q9: Were appropriate methods used for combining or comparing results across studies?	Q10: Do the data support the author's interpretation?	Total quality score
Bakker et al (2020) (7)	1	1	0	1	1	1	1	1	0	1	8 = strong
Cloostermans et al (2015) (46)	1	1	0	1	1	1	1	1	1	1	9 = strong
Dreison et al (2018) (34)	1	1	1	1	1	1	0	1	1	1	9 = strong
Gayed et al (2018) (21)	1	1	1	1	1	1	1	1	1	1	10 = strong
Gilbody et al (2006) (42)	1	1	0	1	1	1	1	1	1	1	9 = strong
Joyce et al (2010) (2)	1	1	1	1	1	1	1	1	1	1	10 = strong
Kuehnl et al (2019) (23)	1	1	1	1	1	1	0	1	1	1	9 = strong
Mimura et al (2003) (53)	1	1	1	1	1	1	0	1	1	1	9 = strong
Missen et al (2014) (11)	1	1	1	1	1	1	0	1	1	1	9 = strong
Naghieh et al (2015) (52)	1	1	1	1	1	1	1	1	1	1	10 = strong
Price et al (2015) (25)	1	1	0	1	1	1	1	1	0	1	8 = strong
Romppanen et al (2017) (43)	1	1	0	1	1	0	1	1	1	1	8 = strong
Ruotsalainen et al (2015) (51)	1	1	1	1	1	1	0	1	1	1	9 = strong
Schalk et al (2010) (16)	1	1	0	1	1	1	1	1	1	1	9 = strong
Spector et al (2016) (31)	1	1	0	1	1	1	0	1	1	1	8 = strong
Stuber et al (2021) (22)	1	1	0	1	1	1	1	1	0	1	8 = strong
Van Laethem et al (2013) (12)	0	0	1	1	1	1	1	1	1	1	8 = strong
van Wyk et al (2010) (45)	1	1	0	1	1	1	0	1	1	1	8 = strong
West et al (2016) (38)	1	1	1	1	1	1	1	1	1	1	10 = strong
Xu et al (2020) (37)	1	1	0	1	1	1	1	1	0	1	8 = strong
Number of reviews with "yes"	19	19	10	20	20	19	13	20	16	20	176
Average across the 20 reviews with strong quality	0,95	0,95	0,5	1	1	0,95	0,65	1	0,80	1	8,8

Quality assessment question	Q1	Q2	Q3	Q4	Q5	Q6	Q7	Q8	Q9	Q10	Total quality score
**Author (year)**											
Anderson et al (2010) (26)	1	1	1	1	1	0	0	1	0	0	6 = moderate
Aust et al (2004) (13)	1	1	0	1	1	0	0	1	1	1	7 = moderate
Avolio et al (2009) (24)	1	1	1	1	0	0	0	1	1	1	7 = moderate
Awa et al (2010) (33)	1	1	0	1	1	0	0	1	0	0	5 = moderate
Bambra et al (2008) (4)	1	1	1	1	1	0	0	1	0	0	6 = moderate
Bambra et al (2008b) (5)	1	1	1	1	1	1	0	1	0	0	7 = moderate
Bambra et al (2007) (14)	1	1	1	1	1	1	0	1	0	0	7 = moderate
Barbosa et al (2014) (29)	1	1	0	1	1	1	0	1	0	1	7 = moderate
Brook et al (2019) (8)	1	1	0	1	1	0	1	1	0	0	6 = moderate
Chen et al (2014) (6)	1	1	0	1	1	0	0	1	0	0	5 = moderate
Collins et al (2004) (19)	1	1	1	1	1	0	0	0	1	0	6 = moderate
Corbiere et al (2009) (41)	1	1	0	0	1	0	1	1	0	0	5 = moderate
Daniels et al (2017) (17)	1	1	0	1	1	0	0	1	1	0	6 = moderate
DeChant et al (2019) (36)	1	1	0	1	1	1	1	0	0	0	6 = moderate
Edwards et al (2015) (10)	1	1	1	1	1	0	1	1	0	0	7 = moderate
Egan et al (2007) (15)	1	1	1	1	1	0	0	1	0	0	6 = moderate
Elliott et al (2012) (30)	1	1	0	1	1	0	0	1	1	1	7 = moderate
Giga et al (2003) (50)	1	1	0	1	1	0	0	1	1	1	7 = moderate
Grover et al (2016) (20)	0	1	0	1	1	0	0	1	1	0	5 = moderate
Kynoch et al (2011) (28)	1	1	1	1	1	0	1	0	0	0	6 = moderate
Lartey et al (2014) (47)	1	1	0	1	1	0	0	1	1	1	7 = moderate
Lee et al (2014) (44)	1	1	0	1	1	1	0	1	0	0	6 = moderate
Montano et al (2014) (40)	0	0	1	1	1	1	0	1	0	1	6 = moderate
Nijp et al (2012) (3)	1	1	0	1	1	0	0	1	0	0	5 = moderate
Paguio et al (2020) (18)	1	1	0	1	1	0	0	1	1	0	6 = moderate
Panagioti et al (2017) (32)	1	1	0	1	1	0	1	1	1	0	7 = moderate
Pijpker et al (2020) (35)	1	1	0	1	1	1	0	1	0	1	7 = moderate
Richardson et al (2008) (48)	1	1	1	1	1	0	0	1	1	0	7 = moderate
Tölli et al (2017) (27)	1	1	0	1	1	1	1	1	0	0	7 = moderate
van der Klink et al (2001) (49)	1	1	1	1	1	0	0	0	1	0	6 = moderate
Williams et al (2018) (39)	0	1	0	1	1	0	1	1	1	0	6 = moderate
Zhang et al (2016) (9)	0	1	0	1	1	0	1	1	1	0	6 = moderate
Number of reviews with ”yes”	28	31	12	31	31	8	9	28	14	8	200
Average across the 32 reviews with moderate quality	0,88	0,97	0,38	0,97	0,97	0,25	0,28	0,85	0,44	0,25	6,25

Quality assessment question	Q1	Q2	Q3	Q4	Q5	Q6	Q7	Q8	Q9	Q10	Total quality score
**Author (year)**											
Anderson et al (2012) (115)	1	1	0	1	1	0	0	0	0	0	4 = weak
Barrientos-Trigo et al (2018) (116)	1	1	0	0	1	0	0	1	0	0	4 = weak
Buykx et al (2010) (117)	1	1	1	1	0	0	0	0	0	0	4 = weak
Brinkert (2010) (118)	1	0	0	1	0	0	0	0	0	0	2 = weak
Brown et al (2017) (119)	1	1	0	1	0	1	0	0	0	0	4 = weak
Busireddy et al (2017) (120)	0	0	0	1	1	0	0	1	0	0	3 = weak
Caulfield et al (2004) (121)	1	0	0	1	1	0	0	1	0	0	4 = weak
Curtis et al (2008) (122)	1	1	0	1	1	0	0	0	0	0	4 = weak
Edwards et al (2002) (123)	1	1	1	1	0	0	0	0	0	0	4 = weak
Escartin (2016) (124)	0	1	0	1	1	0	0	0	0	0	3 = weak
Fothergill et al (2004) (125)	1	0	1	1	0	0	0	0	0	0	3 = weak
Frich et al (2015) (78)	1	0	0	1	1	0	0	1	0	0	4 = weak
Heckemann et al (2015) (126)	0	0	0	1	1	0	0	1	0	1	4 = weak
Johnson et al. (2018) (127)	1	0	1	0	0	0	0	0	0	0	2 = weak
Lees et al (2019) (128)	1	1	0	1	0	0	0	0	0	0	3 = weak
Milner et al (2015) (129)	1	1	1	1	0	0	0	0	0	0	4 = weak
Nastasia et al (2014) (130)	1	1	0	1	1	0	0	0	0	0	4 = weak
Paal et al (2015) (131)	1	1	0	1	0	0	0	0	0	0	3 = weak
Salt et al (2008) (132)	1	0	0	1	1	0	0	0	0	0	3 = weak
Schaufeli et al (2000) (133)	1	0	0	1	0	0	0	0	0	0	2 = weak
Sirola-Karvinen et al (2006) (134)	1	1	1	0	0	0	0	0	0	0	3 = weak
Viselita et al (2019) (111)	1	1	0	0	1	0	0	0	0	0	3 = weak
Wassell (2009) (135)	1	1	0	1	1	0	0	0	0	0	4 = weak
Westermann et al (2014) (136)	1	1	0	1	1	0	0	0	0	0	4 = weak
Number of reviews with ”yes”	21	15	6	20	13	1	0	5	0	0	82
Average across the 24 reviews with weak quality	0.88	0.63	0.25	0.83	0.54	0,04	0	0.21	0	0	3.41

References

1. Aust B, Jakobsen LM, Jaspers SØ, Jørgensen A, Nordentoft M, Jensen AB. Psykosociale arbejdsmiljøinterventioner – hvad virker? En systematisk gennemgang af forskning i interventioner rettet mod det psykosociale arbejdsmiljø samt en oversigt over viden om implementering af arbejdspladsinterventioner. (psychosocial workplace interventions – what works? A systematic overview of research in interventions to improve the psychosocial work environment and an overview of knowledge about implementation of work place interventions.). 2017. https://nfa.dk/da/Forskning/Udgivelse?journalId=6d3eaccf-e2e0-41b0-aaaf-378424dafcc2.
2. Joyce K, Pabayo R, Critchley JA, Bambra C. Flexible working conditions and their effects on employee health and wellbeing. Cochrane Database Syst Rev. 2010(2):CD008009.
3. Nijp HH, Beckers DG, Geurts SA, Tucker P, Kompier MA. Systematic review on the association between employee worktime control and work-non-work balance, health and well-being, and job-related outcomes. Scand J Work Environ Health. 2012;38(4):299-313.
4. Bambra CL, Whitehead MM, Sowden AJ, Akers J, Petticrew MP. Shifting schedules: The health effects of reorganizing shift work. Am J Prev Med. 2008;34(5):427-34.
5. Bambra C, Whitehead M, Sowden A, Akers J, Petticrew M. "A hard day's night?" The effects of compressed working week interventions on the health and work-life balance of shift workers: A systematic review. J Epidemiol Community Health. 2008;62(9):764-77.
6. Chen CM, Lou MF. The effectiveness and application of mentorship programmes for recently registered nurses: A systematic review. J Nurs Manag. 2014;22(4):433-42.
7. Bakker EJM, Kox JHAM, Boot CRL, Francke AL, van der Beek AJ, Roelofs PDDM. Improving mental health of student and novice nurses to prevent dropout: A systematic review. J Adv Nurs. 2020;76(10):2494-509.
8. Brook J, Aitken L, Webb R, MacLaren J, Salmon D. Characteristics of successful interventions to reduce turnover and increase retention of early career nurses: A systematic review. Int J Nurs Stud. 2019;91:47-59.
9. Zhang Y, Qian Y, Wu J, Wen F, Zhang Y. The effectiveness and implementation of mentoring program for newly graduated nurses: A systematic review. Nurse Educ Today. 2016;37:136-44.
10. Edwards D, Hawker C, Carrier J, Rees C. A systematic review of the effectiveness of strategies and interventions to improve the transition from student to newly qualified nurse. Int J Nurs Stud. 2015;52(7):1254-68.
11. Missen K, McKenna L, Beauchamp A. Satisfaction of newly graduated nurses enrolled in transition-to-practice programmes in their first year of employment: A systematic review. J Adv Nurs. 2014;70(11):2419-33.
12. Van Laethem M, Beckers DG, Kompier MA, Dijksterhuis A, Geurts SA. Psychosocial work characteristics and sleep quality: A systematic review of longitudinal and intervention research. Scand J Work Environ Health. 2013;39(6):535-49.
13. Aust B, Ducki A. Comprehensive health promotion interventions at the workplace: Experiences with health circles in germany. J Occup Health Psychol. 2004;9:258-70.
14. Bambra C, Egan M, Thomas S, Petticrew M, Whitehead M. The psychosocial and health effects of workplace reorganisation. 2. A systematic review of task restructuring interventions. J Epidemiol Community Health. 2007;61(12):1028-37.
15. Egan M, Bambra C, Thomas S, Petticrew M, Whitehead M, Thomson H. The psychosocial and health effects of workplace reorganisation. 1. A systematic review of organisational-level interventions that aim to increase employee control. J Epidemiol Community Health. 2007;61(11):945-54.
16. Schalk DM, Bijl ML, Halfens RJ, Hollands L, Cummings GG. Interventions aimed at improving the nursing work environment: A systematic review. Implement Sci. 2010;5:34.
17. Daniels K, Watson D, Gedikli C. Well-being and the social environment of work: A systematic review of intervention studies. Int J Environ Res Public Health. 2017;14(8).
18. Paguio JT, Yu DSF, Su JJ. Systematic review of interventions to improve nurses' work environments. J Adv Nurs. 2020;76(10):2471-93.
19. Collins DB, Holton III EF. The effectiveness of managerial leadership development programs: A meta-analysis of studies from 1982 to 2001. Hum Resour Dev Q. 2004;15(2):217-48.
20. Grover S, Furnham A. Coaching as a developmental intervention in organisations: A systematic review of its effectiveness and the mechanisms underlying it. Plos One. 2016;11(7):e0159137.
21. Gayed A, Milligan-Saville JS, Nicholas J, Bryan BT, LaMontagne AD, Milner A, et al. Effectiveness of training workplace managers to understand and support the mental health needs of employees: A systematic review and meta-analysis. Occup Environ Med. 2018;75(6):462-70.

22. Stuber F, Seifried-Dübon T, Rieger MA, Gündel H, Ruhle S, Zipfel S, et al. The effectiveness of health-oriented leadership interventions for the improvement of mental health of employees in the health care sector: A systematic review. Int Arch Occup Environ Health. 2021;94(2):203-20.
23. Kuehnl A, Seubert C, Rehfuess E, von Elm E, Nowak D, Glaser J. Human resource management training of supervisors for improving health and well-being of employees. Cochrane Database Syst Rev. 2019;9(9):CD010905.
24. Avolio BJ, Reichard RJ, Hannah ST, Walumbwa FO, Chan A. A meta-analytic review of leadership impact research: Experimental and quasi-experimental studies. Leadership Q. 2009;20(5):764-84.
25. Price O, Baker J, Bee P, Lovell K. Learning and performance outcomes of mental health staff training in de-escalation techniques for the management of violence and aggression. Br J Psychiatry. 2015;206(6):447-55.
26. Anderson L, FitzGerald M, Luck L. An integrative literature review of interventions to reduce violence against emergency department nurses. J Clin Nurs. 2010;19(17-18):2520-30.
27. Tölli S, Partanen P, Kontio R, Häggman-Laitila A. A quantitative systematic review of the effects of training interventions on enhancing the competence of nursing staff in managing challenging patient behaviour. J Adv Nurs. 2017;73(12):2817-31.
28. Kynoch K, Wu C-J, Chang AM. Interventions for preventing and managing aggressive patients admitted to an acute hospital setting: A systematic review. Worldviews Evid Based Nurs. 2011;8(2):76-86.
29. Barbosa A, Sousa L, Nolan M, Figueiredo D. Effects of person-centered care approaches to dementia care on staff: A systematic review. Am J Alzheimers Dis Other Demen. 2014.
30. Elliott KE, Scott JL, Stirling C, Martin AJ, Robinson A. Building capacity and resilience in the dementia care workforce: A systematic review of interventions targeting worker and organizational outcomes. Int Psychogeriatr. 2012;24(6):882-94.
31. Spector A, Revolta C, Orrell M. The impact of staff training on staff outcomes in dementia care: A systematic review. Int J Geriatr Psychiatry. 2016;31(11):1172-87.
32. Panagioti M, Panagopoulou E, Bower P, Lewith G, Kontopantelis E, Chew-Graham C, et al. Controlled interventions to reduce burnout in physicians: A systematic review and meta-analysis. JAMA Intern Med. 2017;177(2):195-205.
33. Awa WL, Plaumann M, Walter U. Burnout prevention: A review of intervention programs. Patient Educ Couns. 2010;78(2):184-90.
34. Dreison KC, Luther L, Bonfils KA, Sliter MT, McGrew JH, Salyers MP. Job burnout in mental health providers: A meta-analysis of 35 years of intervention research. J Occup Health Psychol. 2018;23(1):18.
35. Pijpker R, Vaandrager L, Veen EJ, Koelen MA. Combined interventions to reduce burnout complaints and promote return to work: A systematic review of effectiveness and mediators of change. Int J Environ Res Public Health. 2020;17(1):55.
36. DeChant PF, Acs A, Rhee KB, Boulanger TS, Snowdon JL, Tutty MA, et al. Effect of organization-directed workplace interventions on physician burnout: A systematic review. Mayo Clin Proc Innov Qual Outcomes. 2019;3(4):384-408.
37. Xu H, Kynoch K, Tuckett A, Eley R. Effectiveness of interventions to reduce emergency department staff occupational stress and/or burnout: A systematic review. JBI Evid Synth. 2020;18(6):1156-88.
38. West CP, Dyrbye LN, Erwin PJ, Shanafelt TD. Interventions to prevent and reduce physician burnout: A systematic review and meta-analysis. The Lancet. 2016;388(10057):2272-81.
39. Williams SP, Malik HT, Nicolay CR, Chaturvedi S, Darzi A, Purkayastha S. Interventions to improve employee health and well-being within health care organizations: A systematic review. J Healthc Risk Manag. 2018;37(4):25-51.
40. Montano D, Hoven H, Siegrist J. Effects of organisational-level interventions at work on employees' health: A systematic review. BMC Public Health. 2014;14:135.
41. Corbiere M, Shen J, Rouleau M, Dewa CS. A systematic review of preventive interventions regarding mental health issues in organizations. Work. 2009;33(1):81-116.
42. Gilbody S, Cahill J, Barkham M, Richards D, Bee P, Glanville J. Can we improve the morale of staff working in psychiatric units? A systematic review. J Ment Health. 2006;15(1):7-17.
43. Romppanen J, Häggman-Laitila A. Interventions for nurses’ well-being at work: A quantitative systematic review. J Adv Nurs. 2017;73(7):1555-69.
44. Lee NK, Roche A, Duraisingam V, Fischer JA, Cameron J. Effective interventions for mental health in male-dominated workplaces. Ment Health Rev J. 2014;19(4):237-50.

45. van Wyk BE, Pillay-Van W, V. Preventive staff-support interventions for health workers. Cochrane Database Syst Rev. 2010(3):CD003541.
46. Cloostermans L, Bekkers MB, Uiters E, Proper KI. The effectiveness of interventions for ageing workers on (early) retirement, work ability and productivity: A systematic review. Int Arch Occup Environ Health. 2015;88(5):521-32.
47. Lartey S, Cummings G, Profetto-McGrath J. Interventions that promote retention of experienced registered nurses in health care settings: A systematic review. J Nurs Manag. 2014;22(8):1027-41.
48. Richardson KM, Rothstein HR. Effects of occupational stress management intervention programs: A meta-analysis. J Occup Health Psychol. 2008;13(1):69-93.
49. van der Klink JJL, Blonk RWB, Schene AH, van Dijk FJH. The benefits of interventions for work-related stress. Am J Public Health. 2001;91:270-6.
50. Giga SI, Noblet AJ, Faragher B, Cooper CL. The uk perspective: A review of research on organisational stress management interventions. Aust Psychol. 2003;38(2):158-64.
51. Ruotsalainen JH, Verbeek JH, Marine A, Serra C. Preventing occupational stress in healthcare workers. Cochrane Database Syst Rev. 2015;2015(4):CD002892.
52. Naghieh A, Montgomery P, Bonell CP, Thompson M, Aber JL. Organisational interventions for improving wellbeing and reducing work-related stress in teachers. Cochrane Database Syst Rev. 2015;8(4):CD010306.
53. Mimura C, Griffiths P. The effectiveness of current approaches to workplace stress management in the nursing profession: An evidence based literature review. Occup Environ Med. 2003;60(1):10-5.
54. Ahola K, Toppinen-Tanner S, Seppänen J. Interventions to alleviate burnout symptoms and to support return to work among employees with burnout: Systematic review and meta-analysis. Burnout Res. 2017;4:1-11.
55. Hayman-White K, Happell B, Charleston R, Ryan R. Transition to mental health nursing through specialist graduate nurse programs in mental health: A review of the literature. Issues Ment Health Nurs. 2007;28(2):185-200.
56. Levett-Jones T, Fitzgerald M. A review of graduate nurse transition programs in australia. Aust J Adv Nurs. 2005;23(2):40-5.
57. Morse G, Salyers MP, Rollins AL, Monroe-DeVita M, Pfahler C. Burnout in mental health services: A review of the problem and its remediation. Adm Policy Ment Health. 2012;39(5):341-52.
58. Park M, Jones CB. A retention strategy for newly graduated nurses: An integrative review of orientation programs. J Nurses Staff Dev. 2010;26(4):142-9.
59. Paris M, Hoge MA. Burnout in the mental health workforce: A review. J Behav Health Serv Res. 2010;37(4):519-28.
60. Thomas NK. Resident burnout. JAMA. 2004;292(23):2880-9.
61. Tsutsumi A. Development of an evidence-based guideline for supervisor training in promoting mental health: Literature review. J Occup Health. 2011;53(1):1-9.
62. Verbeek J, Ruotsalainen J, Laitinen J, Korkiakangas E, Lusa S, Manttari S, et al. Interventions to enhance recovery in healthy workers; a scoping review. Occup Med (Lond). 2019;69(1):54-63.
63. Weissbrodt R, Giauque D. Labour inspections and the prevention of psychosocial risks at work: A realist synthesis. Safety Sci. 2017;100:110-24.
64. Zhang XJ, Song YQ, Jiang TT, Ding N, Shi TY. Interventions to reduce burnout of physicians and nurses an overview of systematic reviews and meta-analyses. Medicine (Baltimore). 2020;99(26):e20992.
65. Alilyyani B, Wong CA, Cummings G. Antecedents, mediators, and outcomes of authentic leadership in healthcare: A systematic review. Int J Nurs Stud. 2018;83:34-64.
66. Cummings GG, Tate K, Lee S, Wong CA, Paananen T, Micaroni SPM, et al. Leadership styles and outcome patterns for the nursing workforce and work environment: A systematic review. Int J Nurs Stud. 2018;85:19-60.
67. Hall LH, Johnson J, Watt I, Tsipa A, O'Connor DB. Healthcare staff wellbeing, burnout, and patient safety: A systematic review. Plos One. 2016;11(7):e0159015.
68. McVicar A. Workplace stress in nursing: A literature review. J Adv Nurs. 2003;44(6):633-42.
69. Clough BA, March S, Chan RJ, Casey LM, Phillips R, Ireland MJ. Psychosocial interventions for managing occupational stress and burnout among medical doctors: A systematic review. Syst Rev. 2017;6(1):144.
70. Edwards D, Burnard P. A systematic review of stress and stress management interventions for mental health nurses. J Adv Nurs. 2003;42(2):169-200.
71. Maricuţoiu LP, Sava FA, Butta O. The effectiveness of controlled interventions on employees’ burnout: A meta-analysis. J Occup Organ Psychol. 2016;89(1):1-27.

72. Michie S, Williams S. Reducing work related psychological ill health and sickness absence: A systematic literature review. Occup Environ Med. 2003;60(1):3-9.
73. Nowrouzi B, Lightfoot N, Lariviere M, Carter L, Rukholm E, Schinke R, et al. Occupational stress management and burnout interventions in nursing and their implications for healthy work environments: A literature review. Workplace Health Saf. 2015;63(7):308-15.
74. Oprea BT, Barzin L, Virga D, Iliescu D, Rusu A. Effectiveness of job crafting interventions: A meta-analysis and utility analysis. Eur J Work Organ Psychol. 2019;28(6):723-41.
75. Ouellette RR, Goodman AC, Martinez-Pedraza F, Moses JO, Cromer K, Zhao X, et al. A systematic review of organizational and workforce interventions to improve the culture and climate of youth-service settings. Adm Policy Ment Health. 2020;47(5):764-78.
76. Regehr C, Glancy D, Pitts A, LeBlanc VR. Interventions to reduce the consequences of stress in physicians: A review and meta-analysis. J Nerv Ment Dis. 2014;202(5):353-9.
77. Anger WK, Elliot DL, Bodner T, Olson R, Rohlman DS, Truxillo DM, et al. Effectiveness of total worker health interventions. J Occup Health Psychol. 2015;20(2):226.
78. Frich JC, Brewster AL, Cherlin EJ, Bradley EH. Leadership development programs for physicians: A systematic review. J Gen Intern Med. 2015;30(5):656-74.
79. Jones RJ, Woods SA, Guillaume YRF. The effectiveness of workplace coaching: A meta-analysis of learning and performance outcomes from coaching. J Occup Organ Psychol. 2016;89(2):249-77.
80. Knight C, Patterson M, Dawson J. Building work engagement: A systematic review and meta-analysis investigating the effectiveness of work engagement interventions. J Organ Behav. 2017;38(6):792-812.
81. Knight C, Patterson M, Dawson J. Work engagement interventions can be effective: A systematic review. Eur J Work Organ Psychol. 2019;28(3):348-72.
82. Rapaport P, Livingston G, Murray J, Mulla A, Cooper C. Systematic review of the effective components of psychosocial interventions delivered by care home staff to people with dementia. BMJ Open. 2017;7(2).
83. Shultz CG, Holmstrom HL. The use of medical scribes in health care settings: A systematic review and future directions. J Am Board Fam Med. 2015;28(3):371-81.
84. Armstrong N. Management of nursing workplace incivility in the health care settings: A systematic review. Workplace Health Saf. 2018;66(8):403-10.
85. Bartlett L, Martin A, Neil AL, Memish K, Otahal P, Kilpatrick M, et al. A systematic review and meta-analysis of workplace mindfulness training randomized controlled trials. J Occup Health Psychol. 2019;24(1):108-26.
86. Brand SL, Thompson Coon J, Fleming LE, Carroll L, Bethel A, Wyatt K. Whole-system approaches to improving the health and wellbeing of healthcare workers: A systematic review. PLoS One. 2017;12(12):e0188418.
87. Buljac-Samardzic M, Dekker-van Doorn CM, van Wijngaarden JD, van Wijk KP. Interventions to improve team effectiveness: A systematic review. Health Policy. 2010;94(3):183-95.
88. Chesak SS, Cutshall SM, Bowe CL, Montanari KM, Bhagra A. Stress management interventions for nurses: Critical literature review. J Holist Nurs. 2019;37(3):288-95.
89. Cooklin A, Joss N, Husser E, Oldenburg B. Integrated approaches to occupational health and safety: A systematic review. Am J Health Promot. 2017;31(5):401-12.
90. Czabala C, Charzynska K, Mroziak B. Psychosocial interventions in workplace mental health promotion: An overview. Health Promot Int. 2011;26 Suppl 1:i70-i84.
91. De Oliveira SM, de Alcantara Sousa LV, do Socorro Vieira Gadelha M, do Nascimento VB. Prevention actions of burnout syndrome in nurses: An integrating literature review. Clin Pract Epidemiol Ment Health. 2019;15:64-73.
92. Duhoux A, Menear M, Charron M, Lavoie-Tremblay M, Alderson M. Interventions to promote or improve the mental health of primary care nurses: A systematic review. J Nurs Manag. 2017;25(8):597-607.
93. Elder EG, Johnston A, Wallis M, Crilly J. Work-based strategies/interventions to ameliorate stressors and foster coping for clinical staff working in emergency departments: A scoping review of the literature. Australas Emerg Care. 2020;23(3):181-92.
94. Francke AL, de Graaff FM. The effects of group supervision of nurses: A systematic literature review. Int J Nurs Stud. 2012;49(9):1165-79.
95. Kuoppala J, Lamminpaa A, Husman P. Work health promotion, job well-being, and sickness absences--a systematic review and meta-analysis. J Occup Environ Med. 2008;50(11):1216-27.
96. LaMontagne AD, Keegel T, Louie AM, Ostry A, Landsbergis PA. A systematic review of the job-stress intervention evaluation literature, 1990-2005. Int J Occup Environ Health. 2007;13(3):268-80.

97. Martin A, Sanderson K, Cocker F. Meta-analysis of the effects of health promotion intervention in the workplace on depression and anxiety symptoms. Scand J Work Environ Health. 2009;35(1):7-18.
98. McCray LW, Cronholm PF, Bogner HR, Gallo JJ, Neill RA. Resident physician burnout: Is there hope? Fam Med. 2008;40(9):626-32.
99. Meyers MC, van Woerkom M, Bakker AB. The added value of the positive: A literature review of positive psychology interventions in organizations. Eur J Work Organ Psychol. 2013;22(5):618-32.
100. Moran AM, Coyle J, Pope R, Boxall D, Nancarrow SA, Young J. Supervision, support and mentoring interventions for health practitioners in rural and remote contexts: An integrative review and thematic synthesis of the literature to identify mechanisms for successful outcomes. Hum Resour Health. 2014;12:10.
101. Murray M, Murray L, Donnelly M. Systematic review of interventions to improve the psychological well-being of general practitioners. BMC Fam Pract. 2016;17:36.
102. Niskala J, Kanste O, Tomietto M, Miettunen J, Tuomikoski AM, Kyngas H, et al. Interventions to improve nurses' job satisfaction: A systematic review and meta-analysis. J Adv Nurs. 2020;76(7):1498-508.
103. O'Donovan R, McAuliffe E. A systematic review exploring the content and outcomes of interventions to improve psychological safety, speaking up and voice behaviour. BMC Health Serv Res. 2020;20(1):101.
104. Stewart W, Terry L. Reducing burnout in nurses and care workers in secure settings. Nurs Stand. 2014;28(34):37-45.
105. Steinert Y, Naismith L, Mann K. Faculty development initiatives designed to promote leadership in medical education. A beme systematic review: Beme guide no. 19. Med Teach. 2012;34(6):483-503.
106. Suter E, Deutschlander S, Mickelson G, Nurani Z, Lait J, Harrison L, et al. Can interprofessional collaboration provide health human resources solutions? A knowledge synthesis. J Interprof Care. 2012;26(4):261-8.
107. Tan L, Wang MJ, Modini M, Joyce S, Mykletun A, Christensen H, et al. Preventing the development of depression at work: A systematic review and meta-analysis of universal interventions in the workplace. BMC Med. 2014;12:74.
108. Taylor C, Xyrichis A, Leamy MC, Reynolds E, Maben J. Can schwartz center rounds support healthcare staff with emotional challenges at work, and how do they compare with other interventions aimed at providing similar support? A systematic review and scoping reviews. BMJ Open. 2018;8(10).
109. van Mol MM, Kompanje EJ, Benoit DD, Bakker J, Nijkamp MD. The prevalence of compassion fatigue and burnout among healthcare professionals in intensive care units: A systematic review. PLoS One. 2015;10(8):e0136955.
110. Virga D, Maricutoiu LP, Iancu A. The efficacy of work engagement interventions: A meta-analysis of controlled trials. Curr Psychol. 2021;40(12):5863-80.
111. Viselita F, Handiyani H, Pujasari H. Quality level of nursing work life and improvement interventions: Systematic review. Enfermeria Clinica. 2019;29:223-8.
112. Westgaard RH, Winkel J. Occupational musculoskeletal and mental health: Significance of rationalization and opportunities to create sustainable production systems - a systematic review. Appl Ergon. 2011;42(2):261-96.
113. Wiederhold BK, Cipresso P, Pizzioli D, Wiederhold M, Riva G. Intervention for physician burnout: A systematic review. Open Med. 2018;13(1):253-63.
114. De Simone S, Vargas M, Servillo G. Organizational strategies to reduce physician burnout: A systematic review and meta-analysis. Aging Clin Exp Res. 2021;33(4):883-94.
115. Anderson G, Hair C, Todero C. Nurse residency programs: An evidence-based review of theory, process, and outcomes. J Prof Nurs. 2012;28(4):203-12.
116. Barrientos-Trigo S, Vega-Vazquez L, De Diego-Cordero R, Badanta-Romero B, Porcel-Galvez AM. Interventions to improve working conditions of nursing staff in acute care hospitals: Scoping review. J Nurs Manag. 2018;26(2):94-107.
117. Buykx P, Humphreys J, Wakerman J, Pashen D. Systematic review of effective retention incentives for health workers in rural and remote areas: Towards evidence-based policy. Aust J Rural Health. 2010;18(3):102-9.
118. Brinkert R. A literature review of conflict communication causes, costs, benefits and interventions in nursing. J Nurs Manag. 2010;18(2):145-56.
119. Brown D, Igoumenou A, Mortlock AM, Gupta N, Das M. Work-related stress in forensic mental health professionals: A systematic review. J Forensic Pract. 2017;19(3):227-38.
120. Busireddy KR, Miller JA, Ellison K, Ren V, Qayyum R, Panda M. Efficacy of interventions to reduce resident physician burnout: A systematic review. J Grad Med Educ. 2017;9(3):294-301.
121. Caulfield N, Chang D, Dollard MF, Elshaug C. A review of occupational stress interventions in australia. Inter J Stress Manag. 2004;11(2):149-66.

122. Curtis EF, Dreachslin JL. Diversity management interventions and organizational performance: A synthesis of current literature. Hum Res Dev Rev. 2008;7(1):107-34.
123. Edwards D, Hannigan B, Fothergill A, Burnard P. Stress management for mental health professionals: A review of effective techniques. Stress and Health. 2002;18(5):203-15.
124. Escartin J. Insights into workplace bullying: Psychosocial drivers and effective interventions. Psychol Res Behav Manag. 2016;9:157-69.
125. Fothergill A, Edwards D, Burnard P. Stress, burnout, coping and stress management in psychiatrists: Findings from a systematic review. Int J Soc Psychiatry. 2004;50(1):54-65.
126. Heckemann B, Zeller A, Hahn S, Dassen T, Schols JM, Halfens RJ. The effect of aggression management training programmes for nursing staff and students working in an acute hospital setting. A narrative review of current literature. Nurse Educ Today. 2015;35(1):212-9.
127. Johnson J, Hall LH, Berzins K, Baker J, Melling K, Thompson C. Mental healthcare staff well-being and burnout: A narrative review of trends, causes, implications, and recommendations for future interventions. Int J Ment Health Nurs. 2018;27(1):20-32.
128. Lees T, Elliott JL, Gunning S, Newton PJ, Rai T, Lal S. A systematic review of the current evidence regarding interventions for anxiety, ptsd, sleepiness and fatigue in the law enforcement workplace. Ind Health. 2019;57(6):655-67.
129. Milner A, Page K, Spencer-Thomas S, Lamotagne AD. Workplace suicide prevention: A systematic review of published and unpublished activities. Health Promot Int. 2015;30(1):29-37.
130. Nastasia I, Coutu MF, Tcaciuc R. Topics and trends in research on non-clinical interventions aimed at preventing prolonged work disability in workers compensated for work-related musculoskeletal disorders (wrmsds): A systematic, comprehensive literature review. Disabil Rehabil. 2014;36(22):1841-56.
131. Paal P, Helo Y, Frick E. Spiritual care training provided to healthcare professionals: A systematic review. J Pastoral Care Counsel. 2015;69(1):19-30.
132. Salt J, Cummings GG, Profetto-McGrath J. Increasing retention of new graduate nurses: A systematic review of interventions by healthcare organizations. J Nurs Adm. 2008;38(6):287-96.
133. Schaufeli WB, Peeters MCW. Job stress and burnout among correctional officers: A literature review. Inter J Stress Manag. 2000;7:19-48.
134. Sirola-Karvinen P, Hyrkas K. Clinical supervision for nurses in administrative and leadership positions: A systematic literature review of the studies focusing on administrative clinical supervision. J Nurs Manag. 2006;14(8):601-9.
135. Wassell JT. Workplace violence intervention effectiveness: A systematic literature review. Safety Sci. 2009;47(8):1049-55.
136. Westermann C, Kozak A, Harling M, Nienhaus A. Burnout intervention studies for inpatient elderly care nursing staff: Systematic literature review. Int J Nurs Stud. 2014;51(1):63-71.
